# ADHD and adherence to antihypertensive medication treatment: a multinational cohort study

**DOI:** 10.1186/s12916-026-04714-1

**Published:** 2026-02-20

**Authors:** Honghui Yao, Yiling Zhou, Lin Li, Malcolm B. Gillies, Isabell Brikell, Le Gao, Theresa Wimberley, Tian Xie, Yanli Zhang-James, Aske Astrup, Søren Dalsgaard, Birgitte Dige Semark, Anders Engeland, Stephen V. Faraone, Kari Klungsøyr, Henrik Larsson, Kenneth K. C. Man, Harold Snieder, Ian C. K. Wong, Andrew S. C. Yuen, Helga Zoega, Catharina Hartman, Zheng Chang

**Affiliations:** 1 Department of Medical Epidemiology and Biostatistics, Karolinska Institute, Nobels Väg 12A, 171 65 Stockholm, Sweden; 2 Department of Epidemiology, University of Groningen, University Medical Center Groningen, Groningen, Netherlands; 3 School of Population Health, Faculty of Medicine and Health, University of New South Wales, Sydney, NSW Australia; 4 Department of Global Public Health and Primary Care, University of Bergen, Bergen, Norway; 5 Department of Biomedicine, Aarhus University, Aarhus, Denmark; 6 Department of Pharmacy Administration, School of Pharmacy, Xi’an Jiaotong University, Shaanxi, China; 7 Center for Drug Safety and Policy Research, Xi’an Jiaotong University, Shaanxi, China; 8 Centre for Safe Medication Practice and Research, Department of Pharmacology and Pharmacy, Li Ka Shing Faculty of Medicine, Centre for Safe Medication Practice and Research, The University of Hong Kong, Hong Kong Special Administrative Region, Hong Kong, China; 9 National Centre for Register-Based Research, Aarhus University, Aarhus, Denmark; 10 Centre for Integrated Register-Based Research, Aarhus University, Aarhus, Denmark; 11 Guangzhou National Laboratory, Guangzhou International Bio Island, No. 9 XingDaoHuanBei Road, Guangzhou, 510005 Guangdong Province China; 12 Department of Psychiatry, Interdisciplinary Center of Psychopathology and Emotion Regulation, University of Groningen, University Medical Center Groningen, Groningen, Netherlands; 13 Department of Psychiatry and Behavioral Sciences, Norton College of Medicine at SUNY Upstate Medical University, Syracuse, NY USA; 14 Center for Child and Adolescent Psychiatry, Copenhagen University Hospital – Mental Health Services of the Capital Region, Copenhagen, Denmark; 15 Department of Clinical Medicine, University of Copenhagen, Copenhagen, Denmark; 16 Department of Chronic Diseases, Norwegian Institute of Public Health, Bergen, Norway; 17 Department of Health Promotion, Norwegian Institute of Public Health, Bergen, Norway; 18 School of Medical Sciences, Örebro University, Örebro, Sweden; 19 Research Department of Practice and Policy, School of Pharmacy, University College London, London, UK; 20 Centre for Medicines Optimisation Research and Education, University College London Hospitals NHS Foundation Trust, London, UK; 21 Laboratory of Data Discovery for Health, Hong Kong Special Administrative Region, Hong Kong, China; 22 School of Pharmacy, Medical Sciences Division, Macau University of Science and Technology, Macao Special Administrative Region, Macau, China; 23 Aston Pharmacy School, Aston University, Birmingham, UK; 24 Centre of Public Health Sciences, Faculty of Medicine, University of Iceland, Reykjavik, Iceland

**Keywords:** Antihypertensive agents, ADHD, Medication adherence

## Abstract

**Background:**

Adherence to antihypertensive medication, alongside lifestyle modifications, is fundamental to managing hypertension and reducing the risk of cardiovascular disease. Attention-deficit/hyperactivity disorder (ADHD) is a common neurodevelopmental disorder associated with a range of cardiovascular diseases, including hypertension. ADHD medication has also been associated with hypertension. However, the influence of ADHD and ADHD medication on discontinuation and adherence to antihypertensive treatments is unknown.

**Methods:**

We conducted a multinational cohort study using electronic health databases from seven countries, which included adults who initiated antihypertensive medication between 2010 and 2020. ADHD was identified by a diagnosis of ADHD or dispensation of ADHD medications. The outcomes were (1) time to the first discontinuation of antihypertensive medication and (2) poor adherence, defined as the proportion of days covered (PDC) below 80% during 1-, 2-, and 5-year follow-up periods. We used Cox proportional hazards models and logistic regression to estimate associations, adjusting for age, sex, and calendar year of antihypertensive medication initiation. We pooled results from different countries via random-effects meta-analysis.

**Results:**

We identified 12,174,321 adults who initiated antihypertensive medication during the study period, including 320,691 (2.6%) with ADHD. In the pooled analysis across all countries, ADHD was associated with an increased rate of discontinuation in 5-year follow-up of antihypertensive medication (hazard ratio [HR] 1.14; 95% CI, 1.02–1.27). In age-stratified analyses, ADHD was associated with a higher rate of antihypertensive medication discontinuation in middle-aged (HR, 1.11; 95% CI, 1.01–1.23) and older adults (HR, 1.14; 95% CI, 1.01–1.29), but not in young adults. Individuals with ADHD also had higher odds of poor adherence across 1 year after treatment initiation (odds ratio [OR] 1.45, 95% CI 1.26–1.67) to 5 years (OR 1.64, 95% CI 1.34–2.00). Among those with ADHD, use of ADHD medications was associated with lower odds of poor adherence (1 year OR 0.66, 95% CI 0.60–0.73; 5 years OR 0.58, 95% CI 0.46–0.72).

**Conclusions:**

Adults with ADHD are more likely to discontinue antihypertensive treatment and exhibit poor medication adherence. However, ADHD medication use appears to be associated with better adherence among individuals with ADHD.

**Supplementary Information:**

The online version contains supplementary material available at 10.1186/s12916-026-04714-1.

## Background

Hypertension affects 31% of adults worldwide and contributes to 9.4 million deaths and 212 million disability-adjusted life years (DALYs) annually [1, 2]. It is a well-established risk factor for cardiovascular diseases [2]. Treatment of hypertension is critical for the prevention of CVD, and in that where antihypertensive medication is indicated, medication adherence is crucial [3–5].

Attention-deficit/hyperactivity disorder (ADHD) is a prevalent neurodevelopmental disorder characterized by impaired levels of inattention, hyperactivity, and impulsivity [6]. While onset is assumed to be in childhood, ADHD affects up to 2.5% of adults, with potentially higher prevalence given underdiagnoses in older adults [6]. Emerging evidence suggests that individuals with ADHD have approximately twice the risk of cardiovascular conditions, including hypertension, compared with those without ADHD [7, 8]. The symptoms and impairments of ADHD, particularly difficulties with organization, planning, and self-monitoring, may compromise antihypertensive medication adherence [9]. Factors associated with ADHD, such as lower educational attainment and socioeconomic status [10], are also recognized risk factors for low medication adherence in general [11].

Prescriptions for ADHD medications among adults have increased substantially over the past two decades in many countries worldwide, primarily in high-income countries, with the largest increases observed in North American and Nordic countries [12, 13]. These medications may improve adherence by alleviating core symptoms of ADHD, including inattention and impulsivity [14]. However, ADHD medications are associated with side effects such as elevated blood pressure and increased heart rate, which could potentially undermine medication adherence to antihypertensive treatment [7, 15]. To date, no large-scale studies have examined the influence of ADHD or its medications on antihypertensive medication adherence.

This study aimed to address these gaps by testing the hypothesis that individuals with ADHD are more likely to have poor antihypertensive medication adherence. We conducted a multinational cohort study using individual-level data from seven countries to examine the association between ADHD and antihypertensive treatment discontinuation and to assess how ADHD and ADHD medication use relate to long-term adherence. Pooling data across multiple countries enabled us to increase statistical power, enhance the generalizability of findings, and evaluate whether observed associations were consistent across diverse healthcare systems and geographic regions.

## Methods

### Data sources and study design

This multinational cohort study used individual-level data from seven countries, derived from pseudonymized administrative, insurance claims, clinical, and national register databases. Australia contributed data on 7.4 million adults from New South Wales [16]. Denmark [17], the Netherlands, Norway [17], and Sweden [17] provided national health register data. The Health Improvement Network (THIN) dataset in the UK provided primary care data for 6% of the population, and practices that use the database are broadly representative of practices in the UK for patients’ characteristics [18]. In the US, insurance claims were sourced from the TriNetX Research Network database [19]. Medication use was obtained from dispensation records in each country (except for the UK, where only prescription records were available). For simplicity, all references to medications will be referred to as dispensations hereafter. Data was available from January 1, 2009, to December 31, 2020, with some deviations in Australia (July 1, 2012, to December 31, 2020), Denmark (January 1, 2009, to December 31, 2018), and Norway (January 1, 2009, to December 31, 2019). All data sources received necessary ethical approvals, and informed consent was not required for these routinely collected data (Additional file 1: Note 1).

The study included adults (≥18 years) who initiated antihypertensive treatment, defined as the first dispensation of diuretics, calcium channel blockers (CCBs), or ACE inhibitors/ARBs (ATC codes: C03, C08, C09), following a 1-year washout without prior antihypertensive use. We excluded individuals who died, emigrated, or had a history of major adverse cardiovascular events (MACE) on or before treatment initiation, as well as those with missing data on sex, birth year or month, or ATC codes. Definitions of MACE, death, and emigration are provided in Additional file 1: Tables 1 and 2. This study followed Strengthening the Reporting of Observational Studies in Epidemiology (STROBE) guidelines [20].

### Measures

ADHD was identified using ICD-10 code F90 or equivalent database-specific diagnostic codes (e.g., in the UK) and dispensation of ADHD medication. ADHD was considered a time-dependent variable in primary analysis. The six most commonly prescribed ADHD medications were identified using ATC codes or equivalent database-specific codes mapped to ATC codes (Additional file 1: Table 2).

We examined two outcomes: first, discontinuation of antihypertensive medication within 5 years after initiation and poor adherence. Discontinuation was defined as a gap of more than 120 days between consecutive dispensed prescriptions (90-day prescription length plus a 30-day grace period), consistent with previous literature [21]. In a sensitivity analysis, we applied country-specific permissible prescription lengths to define treatment discontinuation: 30 days for Australia and 60 days for Denmark and the UK. Poor adherence was defined as a proportion of days covered (PDC) less than 80% [22], representing the percentage of days within the follow-up period during which the medication was available [23]. PDC was calculated over 1-, 2-, and 5-year follow-up periods [24].

Covariates included age, sex, and calendar year of antihypertensive medication initiation. In secondary analysis, we also adjusted for other psychiatric comorbidities recorded at or prior to the antihypertensive treatment initiation (ICD codes in Additional file 1: Table 3).

### Statistical analysis

We used a distributed network approach to harmonize data and standardize analyses. A common study plan and analysis script were shared with each site, which conducted analyses locally and returned aggregated results to the Swedish coordinating center to ensure data privacy. The study protocol and analysis plan were preregistered on the Open Science Framework (OSF) (10.17605/OSF.IO/S93CQ).

We used Cox regression to estimate the association between ADHD and antihypertensive discontinuation over 5 years, with follow-up time as the underlying time scale. Follow-up began at first antihypertensive dispensation and ended at death, emigration, MACE, discontinuation, 5 years, or study end, whichever came first.

We used logistic regression to assess the association between ADHD and poor adherence at 1, 2, and 5 years. Observation began at the first antihypertensive dispensation and ended at death, emigration, MACE, the respective time point, or study end, whichever came first.

To examine the association between ADHD medication and antihypertensive treatment adherence, we restricted the analysis to individuals with ADHD before their first antihypertensive dispensation to avoid defining the cohort based on future information. ADHD medication treatment periods were defined as beginning on the dispensation date, lasting for the country-specific 75th percentile of the dispensation interval, stratified by age and sex, allowing a maximum gap of 180 days [25]. Overlapping treatment periods were merged into a continuous treatment period. Poor adherence to antihypertensive treatment was then estimated separately for periods with and without ADHD medication use. Logistic regression was used to assess the association between ADHD medication use (with or without ADHD medication) and poor adherence during that same period.

All models were first adjusted for age, sex, and calendar year at antihypertensive initiation. As psychiatric comorbidities were not necessarily confounders, they were only further adjusted for in secondary analyses.

We pooled country-specific association estimates using random-effects meta-analysis and assessed cross-country heterogeneity using Cochran’s Q test and the* I*^*2*^ statistic, with *I*^*2*^ > 75% indicating significant heterogeneity. Within a random-effects meta-analytic framework, we calculated country-specific random effects using Best Linear Unbiased Predictions (BLUPs) [26]. To account for multiple testing across countries, we applied a Bonferroni correction to the *p*-values associated with the BLUP estimates [26]. This analysis indicated that results from the US significantly deviated from the overall meta-analytic estimates in 27 of 42 analyses and demonstrated high heterogeneity compared with other countries (Additional file 1: Table 4). To evaluate the influence of the US results on the overall pooled estimates, we conducted a post-hoc analysis excluding US data.

All analyses were conducted for the full cohort and stratified by sex and age group. Norway was excluded from analyses involving older adults, as its cohort included individuals only up to age 52 at data linkage. Sensitivity analyses used alternative ADHD definitions (lifetime exposure) and discontinuation definitions (varying prescription lengths). Analyses were performed in R.

## Results

We included 12,174,321 adults who initiated antihypertensive treatment (Additional file 1: Table 5), of whom 320,691 (2.6%) had ADHD (Table 1). Across countries, individuals with ADHD were consistently younger at treatment initiation (median age 36–48 years vs 40–61 years) and more often male (52.6% vs 48.9%) compared with those without ADHD. Psychiatric comorbidities were also more common in the ADHD group. ACEIs/ARBs were the most frequently prescribed antihypertensives in both groups, followed by diuretics in most countries; in the UK, CCBs were the second most common (Table 1).

**Table 1:** Characteristics of the study population by country

	Australia		Denmark		Netherlands		Norway		Sweden		UK		US	
	ADHD	Non-ADHD	ADHD	Non-ADHD	ADHD	Non-ADHD	ADHD	Non-ADHD	ADHD	Non-ADHD	ADHD	Non-ADHD	ADHD	Non-ADHD
Number of individuals	7,758	818,725	10,881	702,954	31,514	1,953,185	3,826	98,246	17,227	927,912	356	404,586	249,129	6,948,022
Age at the first dispensing, years (median, IQR)	40 (29, 49)	56 (46, 67)	43 (34, 53)	59 (49, 69)	48 (38, 58)	61 (51, 70)	36 (29, 42)	40 (34, 45)	42 (32, 50)	61 (51, 70)	39 (29, 49)	58 (48, 68)	45 (33, 56)	58 (48, 67)
Follow-up days (median, IQR)	1,321 (610, 2,200)	1,442 (647, 2,331)	1,711 (793, 2,934)	2,010 (958, 3,238)	1,716 (723, 2,852)	1,906 (883, 3,023)	1,446 (656, 2,451)	1,477 (684, 2,465)	1,526 (651, 2,592)	1,687 (727, 2,802)	1,339 (658, 2,517)	2,160 (966, 3,275)	1,817 (974, 2,792)	1,494 (448, 2,519)
Sex														
Male	4,080 (52.6%)	400,472 (48.9%)	5,216 (48.0%)	335,403 (47.7%)	15,550 (49.3%)	925,647 (47.4%)	1,656 (43.3%)	43,722 (44.5%)	8,670 (51.2%)	433,642 (48.0%)	201 (56.5%)	196,204 (48.5%)	95,655 (38.4%)	3,115,441 (44.9%)
Female	3,678 (47.4%)	418,253 (51.1%)	5,665 (52.1%)	368,551 (52.4%)	15,964 (50.7%)	1,027,538 (52.6%)	2,170 (56.7%)	54,524 (55.5%)	8,256 (48.8%)	470,246 (52.0%)	155 (43.5%)	208,382 (51.5%)	153,474 (61.6%)	3,832,581 (55.2%)
Age categories														
Young adult (18–34)	2,868 (37.0%)	71,188 (8.7%)	2,819 (25.9%)	34,170 (4.9%)	6,193 (19.7%)	90,167 (4.6%)	1,703 (44.5%)	26,170 (26.6%)	5,054 (29.9%)	37,903 (4.2%)	139 (39.0%)	22,397 (5.5%)	68,203 (27.4%)	607,876 (8.8%)
Middle-aged (35–64)	4,582 (59.1%)	502,486 (61.4%)	6,974 (64.1%)	409,300 (58.1%)	20,669 (65.6%)	1,077,379 (55.2%)	2,123 (55.5%)	72,076 (73.4%)	11,427 (67.5%)	499,854 (55.3%)	188 (52.8%)	248,567 (61.4%)	154,560 (62.0%)	4,068,997 (58.6%)
Older adults (≥ 65)	308 (4.0%)	245,051 (29.9%)	1,088 (10.0%)	260,480 (37.0%)	4,652 (14.8%)	785,639 (40.2%)	0 (0.0%)	0 (0.0%)	445 (2.6%)	366,131 (40.5%)	29 (8.2%)	133,622 (33.0%)	26,366 (10.6%)	2,271,149 (32.7%)
Psychiatric comorbidities														
Anxiety	930 (12.0%)	21,490 (2.6%)	1,062 (9.8%)	8,075 (1.2%)	3,389 (10.8%)	34,794 (1.8%)	894 (23.4%)	6,567 (6.7%)	9,277 (54.8%)	64,074 (7.1%)	51 (14.3%)	24,496 (6.1%)	64,045 (25.7%)	568,752 (8.2%)
Autism	69 (0.9%)	420 (0.1%)	214 (2.0%)	568 (0.1%)	969 (3.1%)	3,006 (0.2%)	64 (1.7%)	301 (0.3%)	2,400 (14.2%)	2,404 (0.3%)	21 (5.9%)	456 (0.1%)	25 (< 0.1%)	31 (< 0.1%)
Bipolar disorder	434 (5.6%)	6,037 (0.7%)	335 (3.1%)	3,091 (0.4%)	634 (2.0%)	7,494 (0.4%)	294 (7.7%)	1,255 (1.3%)	2,003 (11.8%)	7,732 (0.9%)	13 (3.7%)	2,442 (0.6%)	12,583 (5.1%)	60,231 (0.9%)
Intelligence disorders	42 (0.5%)	2,005 (0.2%)	164 (1.5%)	1,638 (0.2%)	188 (0.6%)	1,858 (0.1%)	61 (1.6%)	501 (0.5%)	489 (2.9%)	2,667 (0.3%)	14 (3.9%)	2,756 (0.7%)	1,243 (0.5%)	10,255 (0.2%)
Depression	1,235 (15.9%)	27,026 (3.3%)	1,886 (17.3%)	18,922 (2.7%)	4,837 (15.4%)	50,843 (2.6%)	1,009 (26.4%)	8,126 (8.3%)	7,229 (42.7%)	45,439 (5.0%)	168 (47.2%)	100,395 (24.8%)	6,868 (2.8%)	38,236 (0.6%)
Schizophrenia	236 (3.0%)	9,925 (1.2%)	560 (5.2%)	7,448 (1.1%)	394 (1.3%)	9,630 (0.5%)	126 (3.3%)	936 (1.0%)	192 (1.1%)	4,544 (0.5%)	9 (2.5%)	5,211 (1.3%)	6,026 (2.4%)	60,605 (0.9%)
Personality disorder	601 (7.8%)	6,744 (0.8%)	1,151 (10.6%)	4,323 (0.6%)	4,736 (15.0%)	33,864 (1.7%)	429 (11.2%)	2,011 (2.1%)	2,916 (17.2%)	10,307 (1.1%)	22 (6.2%)	2,863 (0.7%)	4,920 (2.0%)	16,173 (0.2%)
Eating disorder	113 (1.5%)	886 (0.1%)	157 (1.4%)	926 (0.1%)	557 (1.8%)	3,580 (0.2%)	58 (1.5%)	761 (0.8%)	913 (5.4%)	5,117 (0.6%)	12 (3.4%)	2,811 (0.7%)	3,315 (1.3%)	11,968 (0.2%)
Substance use disorder	1,176 (15.2%)	37,884 (4.6%)	1,566 (14.4%)	17,596 (2.5%)	2,624 (8.3%)	16,896 (0.9%)	830 (21.7%)	3,933 (4.0%)	5,699 (33.7%)	40,052 (4.4%)	53 (14.9%)	6,010 (1.5%)	31,971 (12.8%)	495,729 (7.1%)
Type of first antihypertensive prescriptions^a^														
ACEI/ARB	4,642 (59.8%)	583,258 (71.2%)	4,655 (42.7%)	343,158 (48.8%)	13,153 (41.7%)	856,487 (43.9%)	1,903 (49.7%)	58,973 (60.0%)	8,215 (47.9%)	520,786 (56.1%)	166 (46.6%)	160,607 (39.7%)	113,333 (45.5%)	3,917,877 (56.4%)
Calcium channel blockers	1,171 (15.1%)	106,426 (13.0%)	2,093 (19.2%)	153,385 (21.8%)	8,495 (27.0%)	436,569 (22.4%)	741 (19.7%)	19,660 (20.0%)	4,409 (25.6%)	222,156 (23.9%)	110 (30.9%)	156,274 (38.6%)	61,847 (24.8%)	2,112,291 (30.4%)
Diuretics	2,056 (26.5%)	144,377 (17.6%)	4,349 (40.0%)	227,641 (32.4%)	10,680 (33.9%)	734,320 (37.6%)	1,275 (33.3%)	22,625 (23.0%)	4,603 (26.7%)	184,970 (19.9%)	87 (24.4%)	96,190 (23.8%)	121,889 (48.9%)	3,016,731 (43.4%)

ACEI/ARB, angiotensin-converting enzyme inhibitors/angiotensin receptor blockers

^a^It is possible that individuals received more than one type of antihypertensives in the first dispensation

### ADHD and the first discontinuation of antihypertensive medication

**Fig. 1:**
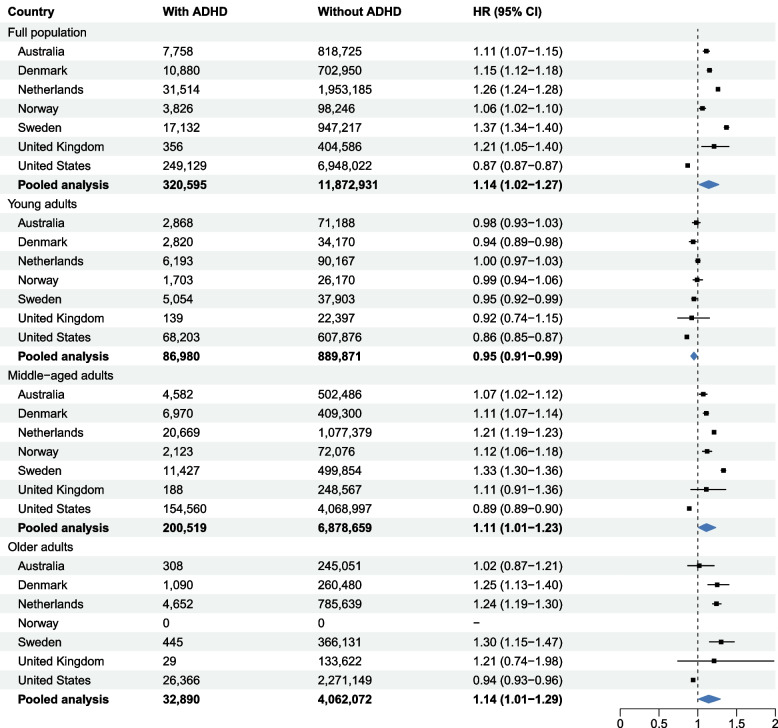
Association between ADHD and first discontinuation of antihypertensive medication. Notes: Models were adjusted for age, sex, and calendar year at the time of first dispensation of antihypertensive medication. In age-stratified analyses, age was not included as an adjustment variable. Abbreviations: ADHD, attention-deficit/hyperactivity disorder; CI, confidence interval; HR, hazard ratio; UK, United Kingdom; US, United States

ADHD was associated with an increased rate of discontinuation in all countries except the US. Meta-analysis showed a 14% increased rate of discontinuation among individuals with ADHD (pooled hazard ratio [HR], 1.14; 95% confidence interval [CI], 1.02–1.27) (Fig. 1). This association was attenuated after adjusting for other psychiatric disorders (HR, 1.07; 95% CI 0.99–1.24; Additional file 1: Table 6). Cross-country heterogeneity was high (*I*^*2*^ > 75; *p* < 0.05). Country-specific HRs ranged from 0.87 (0.87–0.88) in the US to 1.37 (1.34–1.40) in Sweden in the full study population in the specific country (Fig. 1).

In age-stratified meta-analyses, ADHD was associated with higher discontinuation rates among middle-aged (HR, 1.11; 95% CI, 1.01–1.23) and older adults (HR, 1.14; 95% CI, 1.01–1.29), but with a lower rate among young adults (HR, 0.95; 95% CI, 0.91–0.99). These associations attenuated after additional adjustment for psychiatric comorbidities (Additional file 1: Table 6). In post-hoc analyses excluding US data, the association strengthened in the full study population (HR, 1.19; 95% CI, 1.10–1.29), as well as in middle-aged (HR, 1.16; 95% CI, 1.08–1.25) and older adults (HR, 1.24; 95% CI, 1.19–1.28). The association was no longer significant in young adults (HR, 0.97; 95% CI, 0.95–1.00).

### ADHD and poor adherence to antihypertensive medication

**Fig. 2:**
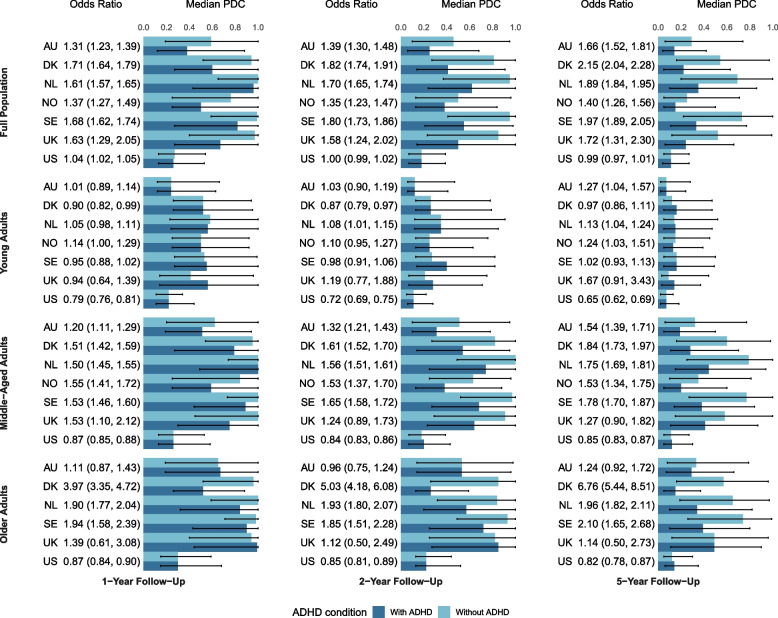
Association between ADHD and poor adherence to antihypertensive medication. Notes: ORs represent the association between ADHD and poor adherence to antihypertensive medication (PDC < 80%). Models were adjusted for age, sex, and calendar year at the time of first dispensation of antihypertensive medication. In age-stratified analyses, age was not included as an adjustment variable. Abbreviations: ADHD, attention-deficit/hyperactivity disorder; CI, confidence interval; OR, odds ratio; UK, United Kingdom; US, United States

Figure 2 shows country-specific median PDCs and ORs for the association between ADHD and poor antihypertensive adherence at 1, 2, and 5 years. In most countries, individuals with ADHD had lower PDCs than those without ADHD, except in the US, where it was similar between groups.

**Fig. 3:**
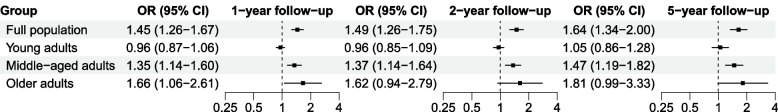
Meta-analysis of the association between ADHD and poor adherence to antihypertensive medication. Models were adjusted for age, sex, and calendar year at the time of first dispensation of antihypertensive medication. In age-stratified analyses, age was not included as an adjustment variable. Abbreviations: ADHD, attention-deficit/hyperactivity disorder; CI, confidence interval; OR, odds ratio; UK, United Kingdom; US, United States

Pooled analyses showed that ADHD was associated with higher odds of poor antihypertensive adherence, except among young adults (Fig. 3). At 1 year after antihypertensive initiation, ADHD was associated with 45% higher odds in the full cohort (OR, 1.45; 95% CI, 1.26–1.67), 35% in middle-aged adults (OR, 1.35; 95% CI, 1.14–1.60), and 66% in older adults (OR, 1.66; 95% CI, 1.06–2.61). By 5 years, the association persisted (full cohort OR, 1.64; 95% CI, 1.34–2.00; middle-aged adults OR, 1.47; 95% CI, 1.19–1.82). Heterogeneity across countries was high (*I*^2^ >75%; *p* < .05). Adjustment for psychiatric comorbidities attenuated estimates, but most associations remained significant (Additional file 1: Tables 7–9). In post-hoc analyses excluding the US, the 5-year associations strengthened in young adults (OR, 1.11; 95% CI, 1.01–1.22) and older adults (OR, 2.16; 95% CI, 1.15–4.03) (Additional file 1: Fig. S2).

### ADHD medication and poor adherence to antihypertensive medication

Among adults with ADHD, the median age at first antihypertensive dispensing ranged from 36 years (Norway) to 48 years (the Netherlands), with a high proportion of males and middle-aged adults in most countries (Additional file 1: Table 10).

**Fig. 4:**
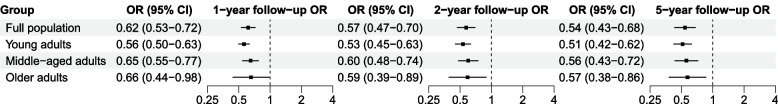
Meta-analyses of the association between ADHD medication and poor adherence to antihypertensive medication among individuals with ADHD. Models were adjusted for age, sex, and calendar year at the time of first dispensation of antihypertensive medication. In age-stratified analyses, age was not included as an adjustment variable. Abbreviations: ADHD, attention-deficit/hyperactivity disorder; CI, confidence interval; OR, odds ratio; UK, United Kingdom; US, United States

Figure 4 shows the association between ADHD medication use and poor adherence to antihypertensive treatment. Overall, ADHD medication use was associated with lower odds of poor adherence. At 1 year post-initiation, ADHD medication use was associated with 38% lower odds of poor adherence (OR 0.62; 95% CI 0.53–0.72) in the full cohort, 44% reduction in young adults (OR 0.56; 95% CI, 0.50–0.63), 35% reduction in middle-aged adults (OR 0.65; 95% CI 0.55–0.77), and 34% reduction in older adults (OR 0.66; 95% CI 0.44–0.98). At 5 years, these differences were 46% in the full study population (OR 0.54; 95% CI 0.43–0.68), 49% in young adults (OR 0.51; 95% CI 0.42–0.62), 44% in middle-aged adults (OR 0.56; 95% CI 0.43–0.72), and 43% in older adults (OR 0.57; 95% CI 0.38–0.86). Cross-country heterogeneity was high (*I*^*2*^ > 75; *p* < 0.05). In post-hoc analyses excluding US data, associations attenuated slightly, but the overall pattern remained (Additional file 1: Fig. S3).

Additional file 1: Table 11 shows that median PDCs were consistently higher during periods covered by ADHD medication. Country-specific estimates (Additional file 1: Tables 12–14) generally supported the pooled estimates, albeit with some heterogeneity. In Denmark and the US, ADHD medication use had the largest association with reduced poor adherence. Among older adults, most countries did not show a clear association at 1 year, except the Netherlands (OR 0.70; 95% CI 0.57–0.85) and the US (OR 0.33; 95% CI 0.31–0.36).

Sex-stratified meta-analyses demonstrated that the association between ADHD and first discontinuation of antihypertensive medication was stronger in males than females, after adjusting for age and calendar year of antihypertensive medication initiation (Additional file 1: Table 6). Similarly, associations between ADHD or ADHD medication use and poor adherence were more pronounced in males (Additional file 1: Tables 7–9, 12–14).

In sensitivity analyses where ADHD was defined as a lifetime exposure, the results were consistent with the main findings (Additional file 1: Tables 15–18). When using country-specific maximum prescription lengths to define discontinuation, associations with first discontinuation remained stable in Australia, Denmark, and the UK (Additional file 1: Tables 19-21). However, the associations between ADHD or ADHD medication and poor adherence moved closer to null compared with the main analyses.

## Discussion

In this study, individuals with ADHD were more likely to discontinue antihypertensive medication and exhibited poorer long-term adherence to antihypertensives, compared to those without ADHD. Among individuals with ADHD, the use of ADHD medication was associated with better adherence to antihypertensive medication treatment compared with non-use.

To our knowledge, no previous study has examined the association between ADHD, ADHD medication use, and adherence to antihypertensive treatment. Our findings suggest that individuals with ADHD are more likely to exhibit lower adherence to antihypertensive medication, which may arise from several factors. First, core ADHD symptoms—such as inattention and impulsivity—can disrupt routine medication-taking behaviors [9]. Second, individuals with ADHD may encounter socioeconomic disadvantage [27], limited health information [28], and communication barriers with healthcare providers [29], all of which may hinder adherence to antihypertensive medication [30]. Third, psychiatric comorbidities and complex treatment regimens [6] can contribute to polypharmacy and potential drug interactions, further complicating adherence. Finally, ADHD medications may have cardiovascular adverse effects [15], and according to clinical guidelines, individuals treated with ADHD medications should be monitored for increased blood pressure [31], prompting earlier detection of hypertension. This may lead to detection bias, as individuals identified during monitoring may perceive their condition as mild or precautionary, reducing motivation to adhere to treatment. Given the strong link between antihypertensive medication adherence and improved cardiovascular outcomes [32], targeted interventions to support adherence in individuals with ADHD are warranted.

ADHD was associated with poorer adherence to antihypertensive medication among middle-aged and older adults. This finding aligns with previous studies that have reported similar associations between depression and poor adherence to antihypertensive treatment in older adults [33]. Several factors might explain this finding. First, middle-aged and older adults often have more cardiovascular comorbidities and complex medication regimens, making adherence more challenging [34]. ADHD-related impairments in executive functioning may exacerbate these challenges in managing multidrug therapies. Second, individuals diagnosed with ADHD later in life may represent a more severely impaired subgroup [6], with persistent symptoms that interfere with health behaviors, including medication adherence. This highlights a potential need for enhanced monitoring and individualized treatment support in older adults with ADHD.

We also observed that ADHD medication use was associated with improved adherence to antihypertensive treatment. One plausible explanation is that treatment with ADHD medication helps mitigate core ADHD symptoms such as inattention and impulsivity [6], thereby supporting consistent medication use. Alternatively, this association may reflect confounding by healthcare engagement. For example, ADHD pharmacotherapy typically involves regular clinical monitoring, including blood pressure checks, which may increase opportunities to detect hypertension and reinforce adherence. Moreover, the process of initiating ADHD pharmacotherapy may prompt clinicians to assess and manage other co-occurring health conditions, including cardiovascular risk, thus increasing the likelihood of initiating and maintaining antihypertensive therapy. Additionally, there may be a correlation in refilling multiple prescriptions during a single pharmacy or clinic visit, which could be improved. We did not observe significant variation in the associations across age groups. One possible explanation is that individuals receiving ADHD medication, regardless of age, are likely to be under more active clinical follow-up, which may mitigate age-related differences in adherence. Moreover, engagement in regular ADHD treatment may reflect greater overall contact with healthcare services and higher health literacy, which could support adherence to other prescribed medications.

The introduction of DSM-5 in 2013, which reduced the symptom threshold for ADHD diagnosis in adults, together with improved recognition of the disorder, has led to an increase in adult ADHD diagnoses [35]. As awareness grows, clinicians managing cardiovascular conditions are more likely to encounter adults with co-occurring ADHD, particularly given the established associations between ADHD and elevated risk of hypertension [1]. This highlights the need for greater clinical attention to ADHD in cardiovascular care settings, where careful coordination of pharmacological treatments may be required to optimize both behavioral and cardiovascular outcomes.

While antihypertensive adherence patterns among individuals with ADHD were largely consistent across participating countries, the US showed a notable deviation. In US data, individuals with ADHD were less likely to discontinue antihypertensive medication than those without ADHD, and overall PDC was lower in the US than in other countries. Several factors may help explain this divergence. First, the US healthcare system is characterized by substantial variability in access to care, insurance coverage, and clinical practices [36]. These differences may influence both the diagnosis and management of ADHD, introducing heterogeneity in treatment patterns and subsequent medication adherence. Second, the decentralized nature of medical records in the US and longitudinal patient records may hinder continuous monitoring and coordinated care, potentially affecting adherence behaviors. We also note that the higher comorbidity rates observed among individuals with ADHD in Sweden, compared with those in Norway and Denmark. In Norway, this may be related to the inclusion of only individuals less than 53 years old, whereas in Denmark, clinicians often record only the mandatory primary diagnosis, while secondary conditions may be underreported [37].

### Limitations

By leveraging real-world prescription data from Europe, Australia, and North America—including nationwide data from several countries—our study findings are likely generalizable to routine clinical practice in these settings. However, important limitations remain. First, we lacked information on prescription indications. Although most ADHD medications included are licensed specifically for ADHD, off-label use can occur; however, the proportion is likely small [25, 38] and unlikely to have materially influenced the results. Similarly, antihypertensives may be prescribed for conditions other than hypertension, with indications varying by age. Second, the observed associations may not be causal, as residual confounding by unmeasured factors may partly explain the association. For example, ethnicity, socioeconomic, marital or family status, which have been shown to influence health-seeking behaviors and medication adherence. Third, physical comorbidities were not included in the analyses. Although such conditions may affect medication adherence, particularly among older individuals with multimorbidity and polypharmacy, they are more likely to lie on the causal pathway between ADHD and antihypertensive adherence and thus act as mediators rather than confounders. Finally, because our study was limited to data from high-income countries, the findings may not generalize to low- and middle-income countries. Further research is needed to examine these associations in other regions.

## Conclusions

This multinational study found that individuals with ADHD were more likely to discontinue antihypertensive medications and had poorer adherence compared with those without ADHD. ADHD medication use was associated with improved adherence. While causality cannot be established, this finding suggests that supporting adherence to ADHD medication may also benefit management of co-occurring conditions such as hypertension. These results underscore the importance of healthcare professionals recognizing the specific challenges individuals with ADHD may face in maintaining consistent medication use. Tailored strategies, such as regular follow-up, patient education, and tools that support routine and organization, may help improve adherence and reduce long-term cardiovascular risk in this population.

## Supplementary Information

Additional File 1: Note 1. Ethical review and data sharing summary. Table 1. Exclusion criteria information by country. Table 2. Codes for ADHD medication and major adverse cardiovascular events (MACE). Table 3. ICD codes used to define other psychiatric comorbidities. Table 4. Countries with significant variation after the Bonferroni correction. Table 5. Exclusion criteria and the number of individuals retained at each step. Table 6. Association between ADHD and first discontinuation of antihypertensive medication treatment. Table 7. Association between ADHD and poor adherence to antihypertensive medication for a 1-year follow-up. Table 8. Association between ADHD and poor adherence to antihypertensive medication for a 2-year follow-up. Table 9. Association between ADHD and poor adherence to antihypertensive medication for a 5-year follow-up. Table 10. Baseline characteristics of adults with ADHD. Table 11. Median and interquartile range of the proportion of days covered at different follow-up times among individuals with ADHD with/without ADHD medication across countries. Table 12. Association between ADHD medication and poor adherence to antihypertensive medication among individuals with ADHD at 1-year follow-up. Table 13. Association between ADHD medication and poor adherence to antihypertensive medication among individuals with ADHD at 2-year follow-up. Table 14. Association between ADHD medication and poor adherence to antihypertensive medication among individuals with ADHD at 5-year follow-up. Table 15. Sensitivity analysis: association between ADHD and first discontinuation of antihypertensive medication treatment after using ADHD as a lifetime exposure. Table 16. Sensitivity analysis: association between ADHD and poor adherence to antihypertensive medication at 1-year follow-up after using ADHD as a lifetime exposure. Table 17. Sensitivity analysis: association between ADHD and poor adherence to antihypertensive medication at 2-year follow-up after using ADHD as a lifetime exposure. Table 18. Sensitivity analysis: association between ADHD and poor adherence to antihypertensive medication at 5-year follow-up after using ADHD as a lifetime exposure. Table 19. Sensitivity analysis: association between ADHD and first discontinuation of antihypertensive medication treatment by using the country-specific gap to define discontinuation. Table 20. Sensitivity analysis: association between ADHD and poor adherence to antihypertensive medication by using the country-specific gap to define discontinuation. Table 21. Sensitivity analysis: association between ADHD medication and poor adherence to antihypertensive medication by using the country-specific gap to define discontinuation. Fig. S1. Association between ADHD and first discontinuation of antihypertensive medication treatment (pooled estimates excluding US results). Fig. S2. Meta-analysis of the association between ADHD and poor adherence to antihypertensive medication (pooled estimates excluding US results). Fig. S3. Meta-analysis of the association between ADHD medication and poor adherence to antihypertensive medication (pooled estimates excluding US results).

## Data Availability

Country-specific regulations and laws prohibit sharing or making the individual-level data in this study publicly available. Access to these data is not possible without the permission of the relevant approving human research ethics committees or the data custodians. Details and country-specific data sharing statements are provided in the supplementary materials.

## Abbreviations

ADHD: Attention-deficit/hyperactivity disorder
BLUPs: Best Linear Unbiased Predictions
CCBs: Calcium channel blockers
CI: Confidence interval
DALYs: Disability-adjusted life years
HR: Hazard ratio
MACE: Major adverse cardiovascular events
OR: Odds ratio
OSF: Open Science Framework
PDC: Proportion of days covered
STROBE: Strengthening the Reporting of Observational Studies in Epidemiology
