# Supplementary material for: ADHD and adherence to antihypertensive medication treatment: a multinational cohort study
Source: BMC Med. 2026 Feb 20;24:122. doi: 10.1186/s12916-026-04714-1 (PMC12930603; doi:10.1186/s12916-026-04714-1)
Supplement: Supplementary file 1 — Additional File 1: Note 1. Ethical review and data sharing summary. Table 1. Exclusion criteria information by country. Table 2. Codes for ADHD medication and major adverse cardiovascular events (MACE). Table 3. ICD codes used to define other psychiatric comorbidities. Table 4. Countries with significant variation after the Bonferroni correction. Table 5. Exclusion criteria and the number of individuals retained at each step. Table 6. Association between ADHD and first discontinuation of antihypertensive medication treatment. Table 7. Association between ADHD and poor adherence to antihypertensive medication for a 1-year follow-up. Table 8. Association between ADHD and poor adherence to antihypertensive medication for a 2-year follow-up. Table 9. Association between ADHD and poor adherence to antihypertensive medication for a 5-year follow-up. Table 10. Baseline characteristics of adults with ADHD. Table 11. Median and interquartile range of the proportion of days covered at different follow-up times among individuals with ADHD with/without ADHD medication across countries. Table 12. Association between ADHD medication and poor adherence to antihypertensive medication among individuals with ADHD at 1-year follow-up. Table 13. Association between ADHD medication and poor adherence to antihypertensive medication among individuals with ADHD at 2-year follow-up. Table 14. Association between ADHD medication and poor adherence to antihypertensive medication among individuals with ADHD at 5-year follow-up. Table 15. Sensitivity analysis: association between ADHD and first discontinuation of antihypertensive medication treatment after using ADHD as a lifetime exposure. Table 16. Sensitivity analysis: association between ADHD and poor adherence to antihypertensive medication at 1-year follow-up after using ADHD as a lifetime exposure. Table 17. Sensitivity analysis: association between ADHD and poor adherence to antihypertensive medication at 2-year follow-up after using AD [file 12916_2026_4714_MOESM1_ESM.docx]

ADHD and adherence to antihypertensive medication treatment: A multinational cohort study

Author: Honghui Yao, MSc, DMD^1^*; Yiling Zhou, MRes^2^*; Malcolm B Gillies, PhD^3^*; Lin Li, PhD^1^*; Isabell Brikell, PhD^1,4,5^; Le Gao, PhD^6,7,8^; Theresa Wimberley, PhD^9,10^; Tian Xie, PhD^11,12^; Yanli Zhang-James, PhD^13^; Aske Astrup, Msc^9,10^; Prof. Søren Dalsgaard, PhD^9,14,15^; Birgitte Dige Semark, Msc^9,10^; Prof. Anders Engeland^4^; Prof. Stephen V. Faraone, PhD^13^; Prof. Kari Klungsøyr, PhD, MD^4,16^; Prof. Henrik Larsson, PhD^1,17^; Kenneth K. C. Man, PhD^8,18,19,20^; Prof. Harold Snieder, PhD^2^; Prof. Ian C. K. Wong, PhD^8,21,22^; Andrew S. C. Yuen, MPharm^18,19^; Prof. Helga Zoega, PhD^+23^; Prof. Catharina Hartman, PhD^+12^; Zheng Chang, PhD^+1^

Contents

[Note 1. Ethical review and data sharing summary 4](#_Toc219714652)

[Table 1. Exclusion criteria information by country 6](#_Toc219714653)

[Table 2. Codes for ADHD medication and major adverse cardiovascular events (MACE) 7](#_Toc219714654)

[Table 3. ICD codes used to define other psychiatric comorbidities 8](#_Toc219714655)

[Table 4. Countries with significant variation after the Bonferroni correlation 9](#_Toc219714656)

[Table 5. Exclusion criteria and the number of individuals retained at each step 10](#_Toc219714657)

[Table 6. Association between ADHD and first discontinuation of antihypertensive medication treatment 11](#_Toc219714658)

[Table 7. Association between ADHD and poor adherence to antihypertensive medication for a 1-year follow-up 13](#_Toc219714659)

[Table 8. Association between ADHD and poor adherence to antihypertensive medication for 2-year follow-up 15](#_Toc219714660)

[Table 9. Association between ADHD and poor adherence to antihypertensive medication for a 5-year follow-up 17](#_Toc219714661)

[Table 10. Baseline Characteristics of Adults With ADHD 19](#_Toc219714662)

[Table 11. Median and interquartile range of the proportion of days covered at different follow-up times among individuals with ADHD with/without ADHD medication across countries 21](#_Toc219714663)

[Table 12. Association between ADHD medication and poor adherence to antihypertensive medication among individuals with ADHD at 1-year follow-up. 23](#_Toc219714664)

[Table 13. Association between ADHD medication and poor adherence to antihypertensive medication among individuals with ADHD at 2-year follow-up. 25](#_Toc219714665)

[Table 14. Association between ADHD medication and poor adherence to antihypertensive medication among individuals with ADHD at 5-year follow-up 27](#_Toc219714666)

[Table 15. Sensitivity analysis: association between ADHD and first discontinuation of antihypertensive medication treatment after using ADHD as a lifetime exposure 29](#_Toc219714667)

[Table 16. Sensitivity analysis: association between ADHD and poor adherence to antihypertensive medication at 1-year follow-up after using ADHD as a lifetime exposure 31](#_Toc219714668)

[Table 17. Sensitivity analysis: association between ADHD and poor adherence to antihypertensive medication at 2-year follow-up after using ADHD as a lifetime exposure 32](#_Toc219714669)

[Table 18. Sensitivity analysis: association between ADHD and poor adherence to antihypertensive medication at 5-year follow-up after using ADHD as a lifetime exposure 33](#_Toc219714670)

[Table 19. Sensitivity analysis: association between ADHD and first discontinuation of antihypertensive medication treatment by using the country-specific gap to define discontinuation 34](#_Toc219714671)

[Table 20. Sensitivity analysis: association between ADHD and poor adherence to antihypertensive medication by using the country-specific gap to define discontinuation 35](#_Toc219714672)

[Table 21. Sensitivity analysis: association between ADHD medication and poor adherence to antihypertensive medication by using the country-specific gap to define discontinuation 37](#_Toc219714673)

[Fig. S1. Association between ADHD and first discontinuation of antihypertensive medication treatment (pooled estimates excluding US results) 39](#_Toc219714674)

[Fig. S2. Meta-analysis of the association between ADHD and poor adherence to antihypertensive medication (pooled estimates excluding US results) 40](#_Toc219714675)

[Fig. S3. Meta-analysis of the association between ADHD medication and poor adherence to antihypertensive medication (pooled estimates excluding US results) 41](#_Toc219714676)

# **Note 1. Ethical review and data sharing summary**

| Country/region | Ethical Review Oversight | Data sharing statement |
| --- | --- | --- |
| Australia | This research received ethical approval from the AIHW Human Research Ethics Committee (AIHW HREC) (approval number EO2021/1/1233) and the NSW Population and Health Services Research Ethics Committee (PHSREC) (approval number 2020/ETH02273). | Direct access to the data and analytical files to other individuals or authorities is not permitted without the express permission of the approving human research ethics committees and data custodians. |
| Denmark | The study was approved by the Danish Data Protection Agency, and data access was agreed by Statistics Denmark and the Danish Health Data Authority. Approval by the Ethics Committee and written informed consent were not required for register-based projects, cf. LBK nr 1338 af 01/09/2020, Act no. 1338 of 1 September 2020, section 10 on research ethics for administration of health scientific research projects and health data scientific research projects. | The data that support the findings of this study are available from Statistics Denmark. The data access required the completion of a detailed application form from the Danish Data Protection Agency, the Danish National Board of Health and Statistics Denmark. Researchers at Danish research institutions may obtain the relevant approval and data. International researchers may gain data access if governed by a Danish research institution having needed approval and data access. For more information on accessing the data, see http://www.dst.dk/. |
| Netherlands | Analyses of administrative health records are exempt from the requirement of informed consent. Individual-level data was anonymized and did not leave the Statistics Netherlands server. | This study was performed under project agreement 9205 microdata services research. Individual-level data is prohibited for sharing or being publicly available. Data can be obtained by application to Statistics Netherlands. |
| Norway | The study was approved by the Regional Ethics Committee in Western Norway (2020/75421). | National regulations and laws prohibit us from sharing or making individual-level data publicly available. The data can be obtained by application to the respective registries and one of the Norwegian Regional Committees for Medical and Health Research Ethics |
| Sweden | This study was approved by the Swedish Ethical Review Authority (2020-06540). Informed consent is not required for pseudo anonymized register-based research according to Swedish law. | The Public Access to Information and Secrecy Act in Sweden prohibits us from making individual-level data publicly available. Researchers who are interested in replicating our work can apply for individual-level data through Statistics Sweden at: https://www.scb.se/en/services/guidance-for-researchers-and-universities/ |
| The United Kingdom | The study was approved by the Health Improvement Network Scientific Review Committee (17THIN102). | Individual-level data is prohibited for sharing or being publicly available. Data could be obtained by application to the Health Improvement Network. |
| The United States | The study was approved by the Institutional Review Board of the SUNY Upstate Medical University (1933232-2). | The individual-level electronic health record data in the TriNetX database cannot be shared, as they are the property of TriNetX and can only be accessed through direct contract with the company. |

# Table 1. Exclusion criteria information by country

|  | **Australia** | **Denmark** | **Netherlands** | **Norway** | **Sweden** | **UK** | **US** |
| --- | --- | --- | --- | --- | --- | --- | --- |
| Death information | √ | √ | √ | √ | √ | √ | √ |
| Emigration information | X | √ | √ | √ | √ | √^*^ | X |
| Major cardiovascular events (MACE) information | √ | √ | √ | √ | √ | X | √ |

**Note:** √ = Exclusion made based on stated criteria; X = exclusion not made, * De-registration in the primary practice.

# Table 2. Codes for ADHD medication and major adverse cardiovascular events (MACE)

| **ADHD medication** | **ATC code** |
| --- | --- |
| ADHD medication | amphetamine [N06BA01], dexamphetamine [N06BA02], methylphenidate [N06BA04], atomoxetine [N06BA09], lisdexamfetamine [N06BA12], and guanfacine [C02AC02] |
| **MACE** | **ICD-10 code** |
| Cerebrovascular diseases and transient ischemic attack | I60-I69, G45 |
| Ischemic heart disease | I20-I24 I25.1- I25.2 I25.5-I25.9 |
| Cardiovascular death | I60-I69, G45, I20-I24 I25.1- I25.2 I25.5-I25.9, I50, I42.0-I42.9 |

# Table 3. ICD codes used to define other psychiatric comorbidities

|  | **ICD-8 Codes** | **ICD-9 Codes** | **ICD-10 Codes** |
| --- | --- | --- | --- |
| **Psychiatric Comorbidities** |  |  |  |
| Anxiety Disorder (Anx) | 300.0 | 300.00,300.02 | F40-F41 |
| Autism spectrum disorders (ASD) | - | 299 | F84 |
| Bipolar Disorder(Bip) | 296.1, 296.3, 296.8 | 296A/ C/D/E/W | F30-F31 |
| Depressive disorder (Dep) | 296.2, 298.0,300.4 | 296B,300E | F32-33 |
| Eating disorders (ED) | 306.5x | 307.5 | F50 |
| Intellectual disability (ID) | 310-315 | 317-319 | F7x |
| Personality disorder (PD) | 301 | 301 | F60, F69 |
| Schizophrenia (SCZ) | 295 except 295.7 | 295A-E/G/W/X | F2x |
| Substance use disorder (SUD) | 291,303,304 | 291,292,304,305A,305X | F10-16,F18-F19 |

# Table 4. Countries with significant variation after the Bonferroni correlation

|  | **ADHD and discontinuation** | **ADHD and poor adherence** | **ADHD medication and poor adherence** |
| --- | --- | --- | --- |
| Full population | US | 1 year: US  2 year: US  5 year: US | 1 year: US  2 year: US  5 year: |
| Male | US | 1 year: US  2 year: US  5 year: US | 1 year: US  2 year:  5 year: |
| Female |  | 1 year: US  2 year: US  5 year: US | 1 year: US  2 year: US  5 year: US |
| Young adults | US | 1 year: US  2 year: US  5 year: US | 1 year:  2 year:  5 year: DK |
| Middle-aged adults | US | 1 year: US  2 year: US  5 year: US | 1 year:  2 year:  5 year: |
| Older adults | US | 1 year: DK  2 year: DK  5 year: DK | 1 year: US  2 year:  5 year: |

Abbreviations: DK, Denmark; US, United States.

# Table 5. Exclusion criteria and the number of individuals retained at each step

|  | **Australia** | **Denmark** | **Netherlands** | **Norway** | **Sweden** | **UK** | **US** |
| --- | --- | --- | --- | --- | --- | --- | --- |
| All individuals using antihypertensive medication | 896,297 | 820,905 | 2,232,107 | 114,845 | 1,187,798 | 407,522 | 7,761,769 |
| After excluding individuals who get MACE diagnosis before or on the same day as receiving antihypertensive medication* | 832,158 | 719,925 | 2,092,082 | 108,520 | 1,187,619 | 407,522 | 7,243,387 |
| After excluding individuals who died or emigrated before or on the same day as receiving ADHD medication* | 831,054 | 717,765 | 2,001,583 | 103,683 | 945,139 | 407,522 | 7,218,031 |
| After excluding individuals with missing values in the birth year and month, sex | 831,050 | 717,485 | 2,001,575 | 103,683 | 945,139 | 407,511 | 7,218,031 |
| After excluding individuals who initiated antihypertensive medication before age 18 | 826,483 | 713,835 | 1,984,699 | 102,072 | 945,139 | 404,942 | 7,197,151 |

**Note**: Exclusion depended on the data available in each country/region, as reported in supplementary table 1.

*Records of emigration prior to ADHD medication dispensation can occur when a person emigrates but is still covered by a country’s healthcare system or when emigration is followed by immigration. Records of death prior to medication dispensation were rare and likely reflected entry errors in the databases.

Abbreviations: ADHD, attention-deficit/hyperactivity disorder; MACE, major adverse cardiovascular events; UK, United Kingdom; US, United States.

# Table 6. Association between ADHD and first discontinuation of antihypertensive medication treatment

| **Country** | **Subgroups** | **Model 1**  **HR (95% CI)** | **Model 2**  **HR (95% CI)** | **Model 3**  **HR (95% CI)** |
| --- | --- | --- | --- | --- |
| Australia | Full group | 1.20 (1.16-1.24) | 1.11 (1.07-1.15) | 1.06 (1.03-1.10) |
|  | Male | 1.15 (1.10-1.20) | 1.12 (1.07-1.17) | 1.08 (1.03-1.14) |
|  | Female | 1.29 (1.23-1.36) | 1.13 (1.07-1.19) | 1.07 (1.02-1.13) |
|  | Young adults | 0.96 (0.91-1.02) | 0.98 (0.93-1.03) | 0.98 (0.93-1.03) |
|  | Middle-aged adults | 1.09 (1.04-1.14) | 1.07 (1.02-1.12) | 1.01 (0.97-1.06) |
|  | Older adults | 0.91 (0.77-1.08) | 1.02 (0.87-1.21) | 0.98 (0.83-1.16) |
| Denmark | Full group | 1.40 (1.37-1.43) | 1.15 (1.12-1.18) | 1.10 (1.08-1.13) |
|  | Male | 1.45 (1.40-1.50) | 1.27 (1.23-1.31) | 1.21 (1.17-1.25) |
|  | Female | 1.36 (1.32-1.41) | 1.07 (1.03-1.10) | 1.03 (1.00-1.07) |
|  | Young adults | 0.95 (0.91-0.99) | 0.94 (0.89-0.98) | 0.95 (0.91-1.00) |
|  | Middle-aged adults | 1.31 (1.27-1.34) | 1.11 (1.07-1.14) | 1.06 (1.03-1.09) |
|  | Older adults | 1.27 (1.14-1.42) | 1.25 (1.13-1.40) | 1.25 (1.12-1.39) |
| Netherlands | Full group | 1.40 (1.38-1.42) | 1.26 (1.24-1.28) | 1.20 (1.18-1.22) |
|  | Male | 1.39 (1.36-1.42) | 1.31 (1.29-1.34) | 1.25 (1.22-1.28) |
|  | Female | 1.43 (1.40-1.46) | 1.23 (1.21-1.26) | 1.18 (1.15-1.20) |
|  | Young adults | 0.96 (0.93-0.99) | 1.00 (0.97-1.03) | 1.01 (0.97-1.04) |
|  | Middle-aged adults | 1.32 (1.29-1.34) | 1.21 (1.19-1.23) | 1.14 (1.12-1.16) |
|  | Older adults | 1.25 (1.19-1.31) | 1.24 (1.19-1.30) | 1.21 (1.15-1.26) |
| Norway | Full group | 1.12 (1.08-1.16) | 1.06 (1.02-1.10) | 1.03 (0.99-1.07) |
|  | Male | 1.14 (1.08-1.21) | 1.08 (1.02-1.15) | 1.03 (0.97-1.10) |
|  | Female | 1.11 (1.05-1.17) | 1.05 (1.00-1.11) | 1.04 (0.98-1.10) |
|  | Young adults | 0.97 (0.91-1.03) | 0.99 (0.94-1.06) | 1.00 (0.94-1.06) |
|  | Middle-aged adults | 1.13 (1.07-1.19) | 1.12 (1.06-1.18) | 1.06 (1.00-1.12) |
| Sweden | Full group | 0.96 (0.95-0.98) | 1.08 (1.06-1.10) | 1.03 (1.01-1.05) |
|  | Male | 0.95 (0.93-0.97) | 1.11 (1.08-1.14) | 1.06 (1.03-1.09) |
|  | Female | 0.99 (0.97-1.02) | 1.08 (1.05-1.11) | 1.03 (1.00-1.06) |
|  | Young adults | 0.88 (0.85-0.91) | 0.94 (0.91-0.97) | 0.96 (0.93-1.00) |
|  | Middle-aged adults | 1.01 (0.99-1.04) | 1.02 (1.00-1.04) | 0.98 (0.95-1.00) |
|  | Older adults | 1.02 (0.91-1.14) | 1.08 (0.97-1.21) | 1.03 (0.92-1.15) |
| UK | Full group | 1.75 (1.55-1.98) | 1.44 (1.27-1.62) | 1.21 (1.07-1.37) |
|  | Male | 1.94 (1.65-2.27) | 1.66 (1.42-1.95) | 1.42 (1.21-1.67) |
|  | Female | 1.62 (1.34-1.95) | 1.29 (1.07-1.55) | 1.06 (0.87-1.27) |
|  | Young adults | 1.11 (0.92-1.33) | 1.19 (0.99-1.43) | 1.17 (0.97-1.40) |
|  | Middle-aged adults | 1.53 (1.29-1.82) | 1.28 (1.07-1.52) | 1.03 (0.86-1.22) |
|  | Older adults | 1.26 (0.80-1.97) | 1.35 (0.86-2.11) | 1.21 (0.77-1.89) |
| US | Full group | 0.98 (0.98-0.99) | 0.87 (0.87-0.87) | 0.87 (0.87-0.88) |
|  | Male | 0.96 (0.95-0.97) | 0.87 (0.86-0.88) | 0.87 (0.87-0.88) |
|  | Female | 1.01 (1.00-1.01) | 0.87 (0.87-0.88) | 0.88 (0.87-0.88) |
|  | Young adults | 0.85 (0.84-0.86) | 0.86 (0.85-0.87) | 0.88 (0.87-0.89) |
|  | Middle-aged adults | 0.88 (0.87-0.88) | 0.89 (0.89-0.90) | 0.90 (0.89-0.90) |
|  | Older adults | 0.93 (0.92-0.94) | 0.94 (0.93-0.96) | 0.93 (0.92-0.95) |
| Pooled analysis (including US results) | Full group | 1.27 (1.13-1.42) | 1.14 (1.02-1.27) | 1.07 (0.99-1.16) |
|  | Male | 1.26 (1.12-1.43) | 1.19 (1.04-1.35) | 1.12 (1.01-1.24) |
|  | Female | 1.29 (1.14-1.45) | 1.11 (1.00-1.23) | 1.05 (0.97-1.13) |
|  | Young adults | 0.92 (0.89-0.96) | 0.95 (0.91-0.99) | 0.96 (0.92-1.00) |
|  | Middle-aged adults | 1.19 (1.04-1.35) | 1.11 (1.01-1.23) | 1.04 (0.97-1.11) |
|  | Older adults | 1.11 (0.97-1.27) | 1.14 (1.01-1.29) | 1.11 (0.99-1.24) |
| Pooled analysis (excluding US results) | Full group | 1.32 (1.21-1.45) | 1.19 (1.10-1.29) | 1.11 (1.06-1.17) |
|  | Male | 1.32 (1.20-1.45) | 1.25 (1.14-1.38) | 1.17 (1.09-1.25) |
|  | Female | 1.35 (1.22-1.49) | 1.16 (1.07-1.26) | 1.09 (1.04-1.14) |
|  | Young adults | 0.94 (0.92-0.97) | 0.97 (0.95-1.00) | 0.98 (0.95-1.00) |
|  | Middle-aged adults | 1.25 (1.14-1.37) | 1.16 (1.08-1.25) | 1.07 (1.03-1.12) |
|  | Older adults | 1.17 (1.03-1.33) | 1.24 (1.19-1.28) | 1.20 (1.15-1.24) |

Model 1 was the crude model; Model 2 adjusted for age, sex, and calendar year at the time of first dispensation of antihypertensive medication; Model 3 additionally adjusted for other psychiatric comorbidities. In age-stratified analyses, age was not included as an adjustment variable.

Abbreviations: ADHD, attention-deficit/hyperactivity disorder; CI, confidence interval; HR, hazard ratio; UK, United Kingdom; US, United States.

# Table 7. Association between ADHD and poor adherence to antihypertensive medication for a 1-year follow-up

| **Country** | **Subgroups** | **Model 1**  **OR (95% CI)** | **Model 2**  **OR (95% CI)** | **Model 3**  **OR (95% CI)** |
| --- | --- | --- | --- | --- |
| Australia | Full group | 1.64 (1.54-1.74) | 1.31 (1.23-1.39) | 1.24 (1.16-1.32) |
|  | Male | 1.47 (1.36-1.59) | 1.27 (1.17-1.37) | 1.22 (1.13-1.32) |
|  | Female | 1.99 (1.80-2.20) | 1.45 (1.31-1.60) | 1.34 (1.21-1.48) |
|  | Young adults | 1.09 (0.97-1.24) | 1.01 (0.89-1.14) | 1.01 (0.90-1.15) |
|  | Middle-aged adults | 1.37 (1.27-1.48) | 1.20 (1.11-1.29) | 1.13 (1.05-1.22) |
|  | Older adults | 1.02 (0.80-1.31) | 1.11 (0.87-1.43) | 1.05 (0.82-1.36) |
| Denmark | Full group | 2.04 (1.95-2.13) | 1.71 (1.64-1.79) | 1.58 (1.51-1.66) |
|  | Male | 2.18 (2.05-2.32) | 2.12 (1.99-2.26) | 1.91 (1.79-2.04) |
|  | Female | 1.95 (1.83-2.08) | 1.47 (1.38-1.57) | 1.38 (1.30-1.48) |
|  | Young adults | 0.90 (0.82-0.99) | 0.90 (0.82-0.99) | 0.95 (0.86-1.05) |
|  | Middle-aged adults | 1.81 (1.71-1.91) | 1.51 (1.42-1.59) | 1.41 (1.33-1.49) |
|  | Older adults | 3.81 (3.22-4.53) | 3.97 (3.35-4.72) | 3.76 (3.17-4.47) |
| Netherlands | Full group | 1.82 (1.77-1.86) | 1.61 (1.57-1.65) | 1.54 (1.50-1.58) |
|  | Male | 1.89 (1.82-1.97) | 1.75 (1.69-1.82) | 1.66 (1.60-1.72) |
|  | Female | 1.83 (1.76-1.89) | 1.52 (1.46-1.58) | 1.46 (1.40-1.51) |
|  | Young adults | 1.02 (0.96-1.09) | 1.05 (0.98-1.11) | 1.06 (1.00-1.13) |
|  | Middle-aged adults | 1.71 (1.66-1.77) | 1.50 (1.45-1.55) | 1.40 (1.36-1.45) |
|  | Older adults | 1.87 (1.74-2.00) | 1.90 (1.77-2.04) | 1.86 (1.73-1.99) |
| Norway | Full group | 1.62 (1.50-1.75) | 1.37 (1.27-1.49) | 1.21 (1.11-1.31) |
|  | Male | 1.70 (1.52-1.89) | 1.40 (1.25-1.56) | 1.16 (1.04-1.31) |
|  | Female | 1.63 (1.46-1.82) | 1.36 (1.22-1.52) | 1.26 (1.12-1.41) |
|  | Young adults | 1.14 (1.01-1.29) | 1.14 (1.00-1.29) | 1.08 (0.95-1.23) |
|  | Middle-aged adults | 1.66 (1.50-1.83) | 1.55 (1.41-1.72) | 1.31 (1.18-1.46) |
| Sweden | Full group | 2.28 (2.21-2.36) | 1.71 (1.65-1.77) | 1.24 (1.19-1.29) |
|  | Male | 2.11 (2.02-2.22) | 1.94 (1.85-2.04) | 1.42 (1.34-1.49) |
|  | Female | 2.64 (2.51-2.77) | 1.63 (1.54-1.71) | 1.18 (1.12-1.25) |
|  | Young adults | 0.84 (0.79-0.90) | 0.91 (0.85-0.98) | 0.88 (0.81-0.95) |
|  | Middle-aged adults | 2.00 (1.92-2.09) | 1.62 (1.55-1.69) | 1.14 (1.08-1.19) |
|  | Older adults | 2.00 (1.62-2.46) | 2.17 (1.76-2.67) | 1.72 (1.39-2.12) |
| UK | Full group | 2.01 (1.60-2.53) | 1.63 (1.29-2.05) | 1.52 (1.20-1.92) |
|  | Male | 2.42 (1.79-3.28) | 2.05 (1.51-2.78) | 1.92 (1.42-2.61) |
|  | Female | 1.74 (1.23-2.50) | 1.29 (0.90-1.85) | 1.18 (0.83-1.70) |
|  | Young adults | 0.86 (0.59-1.25) | 0.94 (0.64-1.39) | 1.01 (0.69-1.50) |
|  | Middle-aged adults | 1.84 (1.33-2.54) | 1.53 (1.10-2.12) | 1.38 (1.00-1.92) |
|  | Older adults | 1.25 (0.56-2.75) | 1.39 (0.61-3.08) | 1.32 (0.58-2.93) |
| US | Full group | 1.04 (1.03-1.06) | 1.04 (1.02-1.05) | 1.04 (1.02-1.05) |
|  | Male | 0.97 (0.95-0.99) | 0.97 (0.95-0.99) | 0.97 (0.95-0.99) |
|  | Female | 1.09 (1.07-1.11) | 1.09 (1.07-1.11) | 1.09 (1.07-1.11) |
|  | Young adults | 0.78 (0.75-0.81) | 0.79 (0.76-0.81) | 0.79 (0.76-0.81) |
|  | Middle-aged adults | 0.87 (0.86-0.89) | 0.87 (0.85-0.88) | 0.87 (0.85-0.88) |
|  | Older adults | 0.87 (0.84-0.90) | 0.87 (0.84-0.90) | 0.87 (0.84-0.90) |
| Pooled analysis (including US results) | Full group | 1.70 (1.42-2.03) | 1.45 (1.26-1.67) | 1.31 (1.16-1.48) |
|  | Male | 1.70 (1.37-2.13) | 1.57 (1.27-1.96) | 1.41 (1.16-1.71) |
|  | Female | 1.76 (1.45-2.14) | 1.40 (1.25-1.56) | 1.27 (1.17-1.39) |
|  | Young adults | 0.95 (0.85-1.06) | 0.96 (0.87-1.06) | 0.96 (0.87-1.05) |
|  | Middle-aged adults | 1.54 (1.25-1.90) | 1.35 (1.14-1.60) | 1.20 (1.04-1.38) |
|  | Older adults | 1.59 (1.01-2.50) | 1.66 (1.06-2.61) | 1.56 (1.00-2.42) |
| Pooled analysis (excluding US results) | Full group | 1.84 (1.68-2.01) | 1.54 (1.40-1.70) | 1.37 (1.23-1.52) |
|  | Male | 1.86 (1.64-2.11) | 1.71 (1.44-2.03) | 1.50 (1.26-1.78) |
|  | Female | 1.94 (1.72-2.18) | 1.49 (1.42-1.57) | 1.32 (1.23-1.42) |
|  | Young adults | 0.98 (0.89-1.09) | 1.00 (0.93-1.07) | 1.00 (0.94-1.07) |
|  | Middle-aged adults | 1.70 (1.53-1.88) | 1.46 (1.34-1.59) | 1.27 (1.15-1.40) |
|  | Older adults | 1.83 (1.16-2.89) | 1.93 (1.24-3.00) | 1.78 (1.14-2.78) |

Models were adjusted for age, sex, and calendar year at the time of first dispensation of antihypertensive medication. In age-stratified analyses, age was not included as an adjustment variable.

Abbreviations: ADHD, attention-deficit/hyperactivity disorder; CI, confidence interval; OR, odds ratio; UK, United Kingdom; US, United States.

# Table 8. Association between ADHD and poor adherence to antihypertensive medication for 2-year follow-up

| **Country** | **Subgroups** | **Model 1**  **OR (95% CI)** | **Model 2**  **OR (95% CI)** | **Model 3**  **OR (95% CI)** |
| --- | --- | --- | --- | --- |
| Australia | Full group | 1.94 (1.82-2.07) | 1.39 (1.30-1.48) | 1.31 (1.23-1.40) |
|  | Male | 1.72 (1.59-1.87) | 1.32 (1.22-1.44) | 1.28 (1.17-1.39) |
|  | Female | 2.44 (2.19-2.73) | 1.58 (1.42-1.77) | 1.46 (1.31-1.64) |
|  | Young adults | 1.24 (1.08-1.42) | 1.03 (0.90-1.19) | 1.05 (0.91-1.21) |
|  | Middle-aged adults | 1.66 (1.53-1.80) | 1.32 (1.21-1.43) | 1.24 (1.15-1.35) |
|  | Older adults | 0.96 (0.75-1.24) | 0.96 (0.75-1.24) | 0.90 (0.70-1.16) |
| Denmark | Full group | 2.31 (2.20-2.42) | 1.82 (1.74-1.91) | 1.66 (1.59-1.75) |
|  | Male | 2.49 (2.33-2.65) | 2.22 (2.08-2.37) | 1.99 (1.86-2.12) |
|  | Female | 2.18 (2.04-2.34) | 1.55 (1.45-1.66) | 1.45 (1.36-1.56) |
|  | Young adults | 0.91 (0.82-1.01) | 0.87 (0.79-0.97) | 0.90 (0.81-1.01) |
|  | Middle-aged adults | 2.04 (1.93-2.16) | 1.61 (1.52-1.70) | 1.49 (1.41-1.58) |
|  | Older adults | 4.67 (3.89-5.64) | 5.03 (4.18-6.08) | 4.77 (3.96-5.77) |
| Netherlands | Full group | 1.85 (1.80-1.90) | 1.70 (1.65-1.74) | 1.65 (1.61-1.69) |
|  | Male | 2.01 (1.94-2.09) | 1.84 (1.78-1.91) | 1.77 (1.71-1.84) |
|  | Female | 1.79 (1.73-1.86) | 1.57 (1.51-1.63) | 1.53 (1.48-1.59) |
|  | Young adults | 1.10 (1.03-1.18) | 1.08 (1.01-1.15) | 1.10 (1.03-1.18) |
|  | Middle-aged adults | 1.86 (1.80-1.92) | 1.56 (1.51-1.61) | 1.48 (1.44-1.53) |
|  | Older adults | 1.84 (1.72-1.97) | 1.93 (1.80-2.07) | 1.92 (1.79-2.06) |
| Norway | Full group | 1.67 (1.54-1.81) | 1.35 (1.23-1.47) | 1.18 (1.08-1.29) |
|  | Male | 1.75 (1.56-1.96) | 1.40 (1.24-1.57) | 1.19 (1.06-1.35) |
|  | Female | 1.66 (1.47-1.87) | 1.31 (1.15-1.48) | 1.19 (1.05-1.36) |
|  | Young adults | 1.15 (1.01-1.32) | 1.10 (0.95-1.27) | 1.05 (0.91-1.21) |
|  | Middle-aged adults | 1.69 (1.52-1.88) | 1.53 (1.37-1.70) | 1.29 (1.16-1.45) |
| Sweden | Full group | 2.68 (2.59-2.77) | 1.83 (1.77-1.90) | 1.36 (1.31-1.41) |
|  | Male | 2.49 (2.38-2.61) | 2.03 (1.93-2.13) | 1.53 (1.46-1.61) |
|  | Female | 3.07 (2.91-3.23) | 1.76 (1.67-1.86) | 1.30 (1.23-1.38) |
|  | Young adults | 0.98 (0.91-1.05) | 1.00 (0.93-1.08) | 1.00 (0.92-1.09) |
|  | Middle-aged adults | 2.30 (2.20-2.39) | 1.70 (1.63-1.77) | 1.22 (1.17-1.28) |
|  | Older adults | 2.05 (1.68-2.51) | 1.97 (1.60-2.41) | 1.61 (1.31-1.98) |
| UK | Full group | 2.06 (1.63-2.63) | 1.58 (1.24-2.02) | 1.47 (1.16-1.89) |
|  | Male | 2.36 (1.74-3.23) | 1.94 (1.43-2.67) | 1.83 (1.34-2.51) |
|  | Female | 1.90 (1.32-2.78) | 1.31 (0.90-1.92) | 1.19 (0.82-1.76) |
|  | Young adults | 1.16 (0.76-1.82) | 1.19 (0.77-1.88) | 1.27 (0.82-2.02) |
|  | Middle-aged adults | 1.60 (1.16-2.22) | 1.24 (0.89-1.73) | 1.13 (0.82-1.58) |
|  | Older adults | 1.03 (0.46-2.27) | 1.12 (0.50-2.49) | 1.06 (0.47-2.36) |
| US | Full group | 1.00 (0.99-1.02) | 1.00 (0.99-1.02) | 1.00 (0.99-1.02) |
|  | Male | 0.92 (0.90-0.95) | 0.92 (0.90-0.95) | 0.92 (0.90-0.95) |
|  | Female | 1.06 (1.04-1.09) | 1.06 (1.04-1.09) | 1.06 (1.04-1.09) |
|  | Young adults | 0.71 (0.68-0.75) | 0.72 (0.69-0.75) | 0.72 (0.69-0.75) |
|  | Middle-aged adults | 0.85 (0.83-0.87) | 0.84 (0.83-0.86) | 0.84 (0.83-0.86) |
|  | Older adults | 0.85 (0.81-0.89) | 0.85 (0.81-0.89) | 0.85 (0.81-0.89) |
| Pooled analysis (including US results) | Full group | 1.83 (1.46-2.29) | 1.49 (1.26-1.75) | 1.35 (1.17-1.56) |
|  | Male | 1.84 (1.43-2.38) | 1.60 (1.26-2.02) | 1.44 (1.17-1.77) |
|  | Female | 1.90 (1.49-2.43) | 1.44 (1.26-1.65) | 1.31 (1.18-1.46) |
|  | Young adults | 1.00 (0.86-1.16) | 0.96 (0.85-1.09) | 0.97 (0.85-1.10) |
|  | Middle-aged adults | 1.64 (1.29-2.09) | 1.37 (1.14-1.64) | 1.23 (1.05-1.43) |
|  | Older adults | 1.59 (0.94-2.70) | 1.62 (0.94-2.79) | 1.53 (0.89-2.61) |
| Pooled analysis (excluding US results) | Full group | 2.03 (1.80-2.30) | 1.60 (1.43-1.79) | 1.43 (1.27-1.60) |
|  | Male | 2.07 (1.82-2.35) | 1.75 (1.47-2.09) | 1.56 (1.32-1.84) |
|  | Female | 2.12 (1.78-2.52) | 1.55 (1.43-1.67) | 1.38 (1.27-1.50) |
|  | Young adults | 1.06 (0.96-1.18) | 1.01 (0.94-1.09) | 1.02 (0.95-1.10) |
|  | Middle-aged adults | 1.87 (1.69-2.08) | 1.52 (1.41-1.64) | 1.33 (1.21-1.45) |
|  | Older adults | 1.83 (1.04-3.20) | 1.86 (1.05-3.33) | 1.73 (0.96-3.12) |

Models were adjusted for age, sex, and calendar year at the time of first dispensation of antihypertensive medication. In age-stratified analyses, age was not included as an adjustment variable.

Abbreviations: ADHD, attention-deficit/hyperactivity disorder; CI, confidence interval; OR, odds ratio; UK, United Kingdom; US, United States.

# Table 9. Association between ADHD and poor adherence to antihypertensive medication for a 5-year follow-up

| **Country** | **Subgroups** | **Model 1**  **OR (95% CI)** | **Model 2**  **OR (95% CI)** | **Model 3**  **OR (95% CI)** |
| --- | --- | --- | --- | --- |
| Australia | Full group | 2.59 (2.38-2.82) | 1.66 (1.52-1.81) | 1.55 (1.42-1.69) |
|  | Male | 2.31 (2.09-2.57) | 1.60 (1.44-1.78) | 1.53 (1.37-1.70) |
|  | Female | 3.22 (2.80-3.73) | 1.87 (1.62-2.18) | 1.70 (1.47-1.98) |
|  | Young adults | 1.83 (1.50-2.25) | 1.27 (1.04-1.57) | 1.27 (1.04-1.58) |
|  | Middle-aged adults | 2.17 (1.97-2.40) | 1.54 (1.39-1.71) | 1.45 (1.31-1.61) |
|  | Older adults | 1.43 (1.07-1.94) | 1.24 (0.92-1.72) | 1.15 (0.85-1.59) |
| Denmark | Full group | 2.73 (2.59-2.88) | 2.15 (2.04-2.28) | 1.97 (1.86-2.08) |
|  | Male | 2.80 (2.61-3.01) | 2.51 (2.33-2.71) | 2.25 (2.09-2.44) |
|  | Female | 2.69 (2.49-2.92) | 1.93 (1.78-2.09) | 1.79 (1.65-1.94) |
|  | Young adults | 1.10 (0.97-1.25) | 0.97 (0.86-1.11) | 0.98 (0.86-1.12) |
|  | Middle-aged adults | 2.43 (2.29-2.59) | 1.84 (1.73-1.97) | 1.70 (1.59-1.81) |
|  | Older adults | 5.42 (4.38-6.79) | 6.76 (5.44-8.51) | 6.50 (5.23-8.19) |
| Netherlands | Full group | 2.04 (1.98-2.09) | 1.89 (1.84-1.95) | 1.89 (1.84-1.95) |
|  | Male | 2.26 (2.18-2.34) | 2.09 (2.01-2.17) | 2.07 (1.99-2.15) |
|  | Female | 1.92 (1.84-2.00) | 1.69 (1.62-1.76) | 1.70 (1.63-1.77) |
|  | Young adults | 1.28 (1.18-1.39) | 1.13 (1.04-1.24) | 1.21 (1.11-1.32) |
|  | Middle-aged adults | 2.13 (2.06-2.20) | 1.75 (1.69-1.81) | 1.72 (1.66-1.78) |
|  | Older adults | 1.75 (1.63-1.88) | 1.96 (1.82-2.11) | 2.00 (1.85-2.15) |
| Norway | Full group | 1.77 (1.60-1.96) | 1.40 (1.26-1.56) | 1.28 (1.14-1.43) |
|  | Male | 1.87 (1.63-2.14) | 1.52 (1.32-1.77) | 1.34 (1.15-1.56) |
|  | Female | 1.72 (1.48-2.02) | 1.30 (1.11-1.53) | 1.24 (1.05-1.47) |
|  | Young adults | 1.41 (1.18-1.70) | 1.24 (1.03-1.51) | 1.19 (0.98-1.45) |
|  | Middle-aged adults | 1.68 (1.49-1.90) | 1.53 (1.34-1.75) | 1.36 (1.19-1.56) |
| Sweden | Full group | 2.97 (2.85-3.08) | 1.94 (1.86-2.03) | 1.54 (1.47-1.61) |
|  | Male | 2.76 (2.62-2.90) | 2.15 (2.03-2.27) | 1.74 (1.64-1.84) |
|  | Female | 3.36 (3.17-3.58) | 1.86 (1.74-1.98) | 1.45 (1.36-1.55) |
|  | Young adults | 1.05 (0.96-1.15) | 0.96 (0.87-1.06) | 0.95 (0.85-1.06) |
|  | Middle-aged adults | 2.60 (2.48-2.72) | 1.78 (1.69-1.86) | 1.37 (1.30-1.44) |
|  | Older adults | 2.62 (2.10-3.29) | 2.10 (1.66-2.67) | 1.97 (1.55-2.51) |
| UK | Full group | 2.33 (1.78-3.08) | 1.72 (1.31-2.30) | 1.65 (1.25-2.20) |
|  | Male | 3.22 (2.25-4.75) | 2.54 (1.76-3.77) | 2.45 (1.70-3.64) |
|  | Female | 1.62 (1.10-2.46) | 1.07 (0.72-1.64) | 1.00 (0.67-1.54) |
|  | Young adults | 1.94 (1.07-3.95) | 1.67 (0.91-3.43) | 1.81 (0.98-3.74) |
|  | Middle-aged adults | 1.81 (1.30-2.57) | 1.27 (0.90-1.82) | 1.20 (0.85-1.73) |
|  | Older adults | 1.03 (0.47-2.43) | 1.14 (0.50-2.73) | 1.09 (0.48-2.63) |
| US | Full group | 0.99 (0.97-1.01) | 0.99 (0.97-1.01) | 0.99 (0.97-1.01) |
|  | Male | 0.88 (0.85-0.91) | 0.88 (0.85-0.91) | 0.88 (0.85-0.91) |
|  | Female | 1.07 (1.04-1.10) | 1.07 (1.04-1.10) | 1.07 (1.04-1.10) |
|  | Young adults | 0.64 (0.61-0.68) | 0.65 (0.62-0.69) | 0.65 (0.62-0.69) |
|  | Middle-aged adults | 0.85 (0.83-0.87) | 0.85 (0.83-0.87) | 0.85 (0.83-0.87) |
|  | Older adults | 0.82 (0.78-0.87) | 0.82 (0.78-0.87) | 0.82 (0.78-0.87) |
| Pooled analysis (including US results) | Full group | 2.07 (1.57-2.73) | 1.64 (1.34-2.00) | 1.51 (1.26-1.81) |
|  | Male | 2.12 (1.54-2.92) | 1.79 (1.35-2.37) | 1.64 (1.27-2.14) |
|  | Female | 2.07 (1.53-2.81) | 1.53 (1.26-1.85) | 1.42 (1.20-1.67) |
|  | Young adults | 1.22 (0.93-1.59) | 1.05 (0.86-1.28) | 1.06 (0.86-1.30) |
|  | Middle-aged adults | 1.85 (1.39-2.45) | 1.47 (1.19-1.82) | 1.35 (1.12-1.63) |
|  | Older adults | 1.80 (1.03-3.13) | 1.81 (0.99-3.33) | 1.75 (0.96-3.20) |
| Pooled analysis (excluding US results) | Full group | 2.35 (2.02-2.74) | 1.80 (1.58-2.04) | 1.63 (1.43-1.87) |
|  | Male | 2.43 (2.13-2.78) | 2.01 (1.70-2.38) | 1.82 (1.53-2.17) |
|  | Female | 2.34 (1.84-2.98) | 1.66 (1.43-1.93) | 1.52 (1.32-1.74) |
|  | Young adults | 1.33 (1.13-1.58) | 1.11 (1.01-1.22) | 1.13 (1.01-1.25) |
|  | Middle-aged adults | 2.15 (1.89-2.43) | 1.69 (1.57-1.81) | 1.49 (1.34-1.66) |
|  | Older adults | 2.15 (1.24-3.70) | 2.16 (1.15-4.03) | 2.06 (1.10-3.89) |

Models were adjusted for age, sex, and calendar year at the time of first dispensation of antihypertensive medication. In age-stratified analyses, age was not included as an adjustment variable.

Abbreviations: ADHD, attention-deficit/hyperactivity disorder; CI, confidence interval; OR, odds ratio; UK, United Kingdom; US, United States.

# Table 10. Baseline Characteristics of Adults With ADHD

|  | **Australia** | **Denmark** | | **Netherlands** | **Norway** | **Sweden** | | **UK** | **US** | |
| --- | --- | --- | --- | --- | --- | --- | --- | --- | --- | --- |
|  | **With ADHD medication** | **With ADHD medication** | **No ADHD medication** | **With ADHD medication** | **With ADHD medication** | **With ADHD medication** | **No ADHD medication** | **With ADHD medication** | **With ADHD medication** | **No ADHD medication** |
| Number of individuals | 4,623 | 6,686 | 305 | 21,383 | 2,784 | 11,105 | 1,337 | 255 | 116,704 | 16,300 |
| Age at the first dispensing, years (median, IQR) | 40 (29, 50) | 43 (33, 51) | 39 (29, 48) | 48 (38, 57) | 36 (29, 42) | 42 (32, 50) | 43 (31, 52) | 38 (27, 49) | 45 (33, 56) | 39 (28, 51) |
| Follow-up days (median, IQR) | 944 (398, 1,708) | 1,449 (717, 2,500) | 1,434 (760, 2,369) | 1,375 (589, 2,443) | 1,141 (503, 2,056) | 1,219 (505, 2,162) | 1,088 (420, 2,141) | 1,121 (532, 2,355) | 1,584 (673, 2,477) | 1,497 (606, 2,306) |
| Sex |  |  |  |  |  |  |  |  |  |  |
| Male | 2,606 (56.4%) | 3,361 (50.3%) | 155 (50.8%) | 10,708 (50.1%) | 1,265 (45.4%) | 5,923 (53.3%) | 724 (54.2%) | 143 (56.1%) | 43,041 (36.9%) | 8,002 (49.1%) |
| Female | 2,017 (43.6%) | 3,325 (49.7%) | 150 (49.2%) | 10,675 (49.9%) | 1,519 (54.6%) | 5,182 (46.7%) | 613 (45.9%) | 112 (43.9%) | 73,663 (63.1%) | 8,298 (50.9%) |
| Age categories |  |  |  |  |  |  |  |  |  |  |
| Young adult (18-34) | 1,708 (37.0%) | 1,856 (27.8%) | 121 (39.7%) | 4,375 (20.5%) | 1,210 (43.5%) | 3,328 (30.0%) | 425 (31.8%) | 111 (43.5%) | 32,114 (27.5%) | 6,676 (41.0%) |
| Middle-aged (35-64) | 2,677 (57.91%) | 4,366 (65.3%) | 176 (57.7%) | 14,214 (66.5%) | 1,574 (56.5%) | 7,475 (67.3%) | 866 (64.8%) | 123 (48.2%) | 73,013 (62.6%) | 8,591 (52.7%) |
| Elderly (≥65) | 238 (5.2%) | 464 (6.9%) | 8 (2.6%) | 2,794 (13.1%) | 0 (0.0%) | 302 (2.7%) | 46 (3.4%) | 21 (8.2%) | 11,577 (9.9%) | 1,033 (6.3%) |
| Anxiety | 607 (13.1%) | 820 (12.3%) | 56 (18.4%) | 2,804 (13.1%) | 696 (25.0%) | 6,106 (55.0%) | 696 (52.1%) | 38 (14.9%) | 33,359 (28.6%) | 7,709 (47.3%) |
| Autism | 60 (1.3%) | 192 (2.9%) | 10 (3.3%) | 886 (4.1%) | 57 (2.1%) | 1,653 (14.9%) | 253 (18.9%) | 17 (6.7%) | 15 (0.0%) | 5 (0.0%) |
| Bipolar disorder | 283 (6.1%) | 258 (3.9%) | 35 (11.5%) | 534 (2.5%) | 244 (8.8%) | 1,517 (13.7%) | 199 (14.9%) | 12 (4.7%) | 6,359 (5.5%) | 2,416 (14.8%) |
| Intelligence disorders | 38 (0.8%) | 135 (2.0%) | 22 (7.2%) | 170 (0.8%) | 58 (2.1%) | 344 (3.1%) | 108 (8.1%) | 12 (4.7%) | 597 (0.5%) | 387 (2.4%) |
| Depression | 783 (16.9%) | 1,441 (21.6%) | 97 (31.8%) | 3,997 (18.7%) | 790 (28.4%) | 5,288 (47.6%) | 599 (44.8%) | 119 (46.7%) | 3,982 (3.4%) | 698 (4.3%) |
| Schizophrenia | 168 (3.6%) | 431 (6.5%) | 50 (16.4%) | 334 (1.6%) | 107 (3.8%) | 135 (1.2%) | 34 (2.5%) | 0 (0.0%) | 2,848 (2.4%) | 1,310 (8.0%) |
| Personality disorder | 381 (8.2%) | 893 (13.4%) | 97 (31.8%) | 3,974 (18.6%) | 345 (12.4%) | 2,131 (19.2%) | 301 (22.5%) | 16 (6.3%) | 2,766 (2.4%) | 914 (5.6%) |
| Eating disorder | 61 (1.3%) | 119 (1.8%) | 7 (2.3%) | 463 (2.2%) | 45 (1.6%) | 634 (5.7%) | 67 (5.0%) | 12 (4.7%) | 1,915 (1.6%) | 448 (2.8%) |
| Substance use disorder | 773 (16.7%) | 1,233 (18.4%) | 93 (30.4%) | 2,257 (10.6%) | 659 (23.7%) | 4,100 (36.9%) | 480 (35.90%) | 45 (17.65%) | 15,300 (13.1%) | 4,955 (30.40%) |
| Type of first antihypertensive* |  |  |  |  |  |  |  |  |  |  |
| ACEI/ARB | 2,836 (61.4%) | 2,648 (39.6%) | 116 (38.0%) | 9,062 (42.4%) | 1,414 (50.8%) | 5,616 (50.6%) | 680 (50.9%) | 61 (23.9%) | 57,703 (49.4%) | 7,089 (43.5%) |
| Calcium channel blockers | 692 (15.0%) | 1,345 (20.1%) | 58 (19.0%) | 6,075 (28.4%) | 570 (20.5%) | 2,639 (23.8%) | 346 (25.9%) | 78 (30.6%) | 28,352 (24.3%) | 4,489 (27.5%) |
| Diuretics | 1,157 (25.0%) | 2,980 (44.6%) | 138 (45.2%) | 6,839 (32.0%) | 869 (31.2%) | 3,038 (27.4%) | 348 (26.0%) | 123 (48.2%) | 57,703 (49.4%) | 7,089 (43.5%) |

*It is possible that individuals with more than one type of antihypertensives in the first dispensation

ACEI/ARB, angiotensin-converting enzyme inhibitors/angiotensin receptor blockers

# Table 11. Median and interquartile range of the proportion of days covered at different follow-up times among individuals with ADHD with/without ADHD medication across countries

| **Country** | **Subgroups** | **1-year follow-up** | | **2-year follow-up** | | **5-year follow-up** | |
| --- | --- | --- | --- | --- | --- | --- | --- |
|  |  | **With ADHD medication** | **No ADHD medication** | **With ADHD medication** | **No ADHD medication** | **With ADHD medication** | **No ADHD medication** |
| Australia | Full group | 0.71(0.21,1.00) | 0.53(0.13,1.00) | 0.62(0.14,1.00) | 0.44(0.10,1.00) | 0.58(0.12,1.00) | 0.40(0.07,1.00) |
|  | Male | 0.83(0.27,1.00) | 0.66(0.21,1.00) | 0.77(0.22,1.00) | 0.59(0.12,1.00) | 0.73(0.19,1.00) | 0.53(0.10,1.00) |
|  | Female | 0.51(0.15,1.00) | 0.41(0.12,1.00) | 0.43(0.10,1.00) | 0.30(0.06,0.95) | 0.39(0.08,0.96) | 0.26(0.05,0.89) |
|  | Young adults | 0.45(0.13,1.00) | 0.35(0.12,0.88) | 0.36(0.08,0.95) | 0.23(0.06,0.78) | 0.28(0.06,0.88) | 0.18(0.04,0.73) |
|  | Middle-aged adults | 0.81(0.28,1.00) | 0.69(0.20,1.00) | 0.74(0.21,1.00) | 0.64(0.14,1.00) | 0.71(0.20,1.00) | 0.59(0.12,1.00) |
|  | Older adults | 0.93(0.47,1.00) | 0.93(0.29,1.00) | 0.93(0.42,1.00) | 0.90(0.24,1.00) | 0.93(0.40,1.00) | 0.85(0.20,1.00) |
| Denmark | Full group | 0.98(0.54,1.00) | 0.81(0.27,1.00) | 0.93(0.42,1.00) | 0.54(0.15,1.00) | 0.87(0.35,1.00) | 0.39(0.10,0.98) |
|  | Male | 0.99(0.59,1.00) | 0.81(0.27,1.00) | 0.94(0.52,1.00) | 0.56(0.18,1.00) | 0.91(0.48,1.00) | 0.43(0.11,0.98) |
|  | Female | 0.97(0.47,1.00) | 0.78(0.27,1.00) | 0.91(0.32,1.00) | 0.52(0.14,1.00) | 0.81(0.24,1.00) | 0.34(0.08,0.96) |
|  | Young adults | 0.85(0.36,1.00) | 0.52(0.26,1.00) | 0.77(0.26,1.00) | 0.26(0.13,0.82) | 0.65(0.17,1.00) | 0.18(0.06,0.58) |
|  | Middle-aged adults | 0.99(0.57,1.00) | 0.87(0.27,1.00) | 0.95(0.49,1.00) | 0.66(0.19,1.00) | 0.91(0.43,1.00) | 0.47(0.12,0.99) |
|  | Older adults | 1.00(0.94,1.00) | 1.00(0.79,1.00) | 1.00(0.90,1.00) | 1.00(0.56,1.00) | 1.00(0.86,1.00) | 1.00(0.48,1.00) |
| Netherlands | Full group | 1.00(0.74,1.00) | 1.00(0.49,1.00) | 1.00(0.51,1.00) | 1.00(0.35,1.00) | 1.00(0.37,1.00) | 0.93(0.23,1.00) |
|  | Male | 1.00(0.97,1.00) | 1.00(0.74,1.00) | 1.00(0.76,1.00) | 1.00(0.49,1.00) | 1.00(0.61,1.00) | 1.00(0.35,1.00) |
|  | Female | 1.00(0.55,1.00) | 1.00(0.47,1.00) | 1.00(0.36,1.00) | 0.87(0.25,1.00) | 1.00(0.24,1.00) | 0.74(0.16,1.00) |
|  | Young adults | 1.00(0.40,1.00) | 0.75(0.24,1.00) | 0.92(0.23,1.00) | 0.49(0.17,1.00) | 0.67(0.11,1.00) | 0.35(0.10,1.00) |
|  | Middle-aged adults | 1.00(0.93,1.00) | 1.00(0.74,1.00) | 1.00(0.67,1.00) | 1.00(0.45,1.00) | 1.00(0.52,1.00) | 1.00(0.32,1.00) |
|  | Older adults | 1.00(0.87,1.00) | 1.00(0.65,1.00) | 1.00(0.78,1.00) | 1.00(0.48,1.00) | 1.00(0.72,1.00) | 0.95(0.43,1.00) |
| Norway | Full group | 0.86(0.34,1.00) | 0.74(0.25,1.00) | 0.76(0.25,1.00) | 0.51(0.14,1.00) | 0.67(0.16,1.00) | 0.44(0.10,1.00) |
|  | Male | 1.00(0.60,1.00) | 0.92(0.38,1.00) | 0.92(0.43,1.00) | 0.77(0.25,1.00) | 0.88(0.35,1.00) | 0.68(0.17,1.00) |
|  | Female | 0.67(0.26,1.00) | 0.50(0.25,1.00) | 0.53(0.17,1.00) | 0.38(0.13,0.98) | 0.44(0.11,0.98) | 0.28(0.07,0.90) |
|  | Young adults | 0.76(0.28,1.00) | 0.51(0.25,1.00) | 0.60(0.18,1.00) | 0.38(0.13,1.00) | 0.48(0.11,1.00) | 0.28(0.08,0.91) |
|  | Middle-aged adults | 0.92(0.43,1.00) | 0.83(0.25,1.00) | 0.86(0.32,1.00) | 0.67(0.20,1.00) | 0.80(0.23,1.00) | 0.62(0.15,1.00) |
| Sweden | Full group | 1.00(0.62,1.00) | 0.96(0.47,1.00) | 0.94(0.43,1.00) | 0.85(0.27,1.00) | 0.87(0.33,1.00) | 0.77(0.21,1.00) |
|  | Male | 1.00(0.80,1.00) | 1.00(0.61,1.00) | 0.99(0.64,1.00) | 0.93(0.43,1.00) | 0.93(0.55,1.00) | 0.86(0.33,1.00) |
|  | Female | 0.94(0.41,1.00) | 0.90(0.25,1.00) | 0.83(0.24,1.00) | 0.71(0.19,1.00) | 0.73(0.16,1.00) | 0.59(0.13,1.00) |
|  | Young adults | 0.94(0.40,1.00) | 0.75(0.24,1.00) | 0.78(0.24,1.00) | 0.58(0.16,1.00) | 0.66(0.15,1.00) | 0.44(0.11,0.98) |
|  | Middle-aged adults | 1.00(0.71,1.00) | 1.00(0.52,1.00) | 0.97(0.56,1.00) | 0.93(0.36,1.00) | 0.93(0.46,1.00) | 0.85(0.29,1.00) |
|  | Older adults | 1.00(0.78,1.00) | 1.00(0.70,1.00) | 0.99(0.53,1.00) | 0.95(0.55,1.00) | 0.93(0.46,1.00) | 0.92(0.48,1.00) |
| UK | Full group | 1.00(0.50,1.00) | 0.93(0.27,1.00) | 1.00(0.46,1.00) | 0.76(0.15,1.00) | 0.57(0.08,1.00) | 1.00(0.49,1.00) |
|  | Male | 1.00(0.88,1.00) | 1.00(0.47,1.00) | 1.00(0.88,1.00) | 0.99(0.27,1.00) | 0.70(0.14,1.00) | 1.00(0.78,1.00) |
|  | Female | 0.93(0.27,1.00) | 0.64(0.15,1.00) | 0.90(0.29,1.00) | 0.46(0.08,1.00) | 0.33(0.06,1.00) | 0.92(0.25,1.00) |
|  | Young adults | 0.87(0.23,1.00) | 0.64(0.14,1.00) | 0.78(0.15,1.00) | 0.47(0.08,1.00) | 0.31(0.06,1.00) | 0.78(0.17,1.00) |
|  | Middle-aged adults | 1.00(0.67,1.00) | 1.00(0.43,1.00) | 1.00(0.66,1.00) | 1.00(0.25,1.00) | 0.93(0.13,1.00) | 1.00(0.77,1.00) |
|  | Older adults | 1.00(1.00,1.00) | 1.00(0.47,1.00) | 1.00(1.00,1.00) | 1.00(0.30,1.00) | 0.92(0.24,1.00) | 1.00(0.99,1.00) |

# Table 12. Association between ADHD medication and poor adherence to antihypertensive medication among individuals with ADHD at 1-year follow-up.

| **Country** | **Subgroups** | **Model 1**  **OR (95% CI)** | **Model 2**  **OR (95% CI)** | **Model 3**  **OR (95% CI)** |
| --- | --- | --- | --- | --- |
| Australia | Full group | 0.77 (0.70-0.85) | 0.75 (0.67-0.83) | 0.75 (0.68-0.83) |
|  | Male | 0.76 (0.66-0.87) | 0.75 (0.65-0.86) | 0.75 (0.65-0.86) |
|  | Female | 0.77 (0.66-0.90) | 0.75 (0.64-0.88) | 0.75 (0.64-0.88) |
|  | Young adults | 0.69 (0.58-0.83) | 0.64 (0.53-0.78) | 0.64 (0.53-0.78) |
|  | Middle-aged adults | 0.82 (0.72-0.94) | 0.80 (0.70-0.91) | 0.80 (0.70-0.92) |
|  | Older adults | 0.93 (0.59-1.47) | 0.91 (0.57-1.45) | 0.95 (0.59-1.51) |
| Denmark | Full group | 0.56 (0.52-0.61) | 0.55 (0.50-0.60) | 0.55 (0.50-0.60) |
|  | Male | 0.57 (0.50-0.64) | 0.56 (0.49-0.63) | 0.55 (0.49-0.63) |
|  | Female | 0.56 (0.50-0.63) | 0.54 (0.48-0.61) | 0.54 (0.48-0.61) |
|  | Young adults | 0.46 (0.39-0.54) | 0.47 (0.40-0.55) | 0.47 (0.40-0.55) |
|  | Middle-aged adults | 0.59 (0.54-0.66) | 0.58 (0.52-0.65) | 0.58 (0.52-0.65) |
|  | Older adults | 0.67 (0.44-1.01) | 0.74 (0.49-1.12) | 0.75 (0.49-1.14) |
| Netherlands | Full group | 0.70 (0.66-0.74) | 0.66 (0.62-0.70) | 0.66 (0.62-0.70) |
|  | Male | 0.72 (0.66-0.79) | 0.69 (0.63-0.75) | 0.69 (0.63-0.75) |
|  | Female | 0.68 (0.63-0.74) | 0.63 (0.58-0.69) | 0.63 (0.58-0.69) |
|  | Young adults | 0.63 (0.56-0.71) | 0.61 (0.54-0.69) | 0.61 (0.54-0.69) |
|  | Middle-aged adults | 0.72 (0.67-0.78) | 0.69 (0.64-0.74) | 0.69 (0.64-0.75) |
|  | Older adults | 0.69 (0.56-0.84) | 0.70 (0.57-0.85) | 0.70 (0.57-0.85) |
| Norway | Full group | 0.72 (0.63-0.82) | 0.72 (0.63-0.83) | 0.72 (0.63-0.83) |
|  | Male | 0.66 (0.54-0.81) | 0.67 (0.54-0.82) | 0.67 (0.54-0.82) |
|  | Female | 0.75 (0.63-0.90) | 0.76 (0.64-0.92) | 0.77 (0.64-0.92) |
|  | Young adults | 0.68 (0.55-0.83) | 0.64 (0.51-0.79) | 0.64 (0.52-0.79) |
|  | Middle-aged adults | 0.78 (0.65-0.93) | 0.78 (0.65-0.93) | 0.79 (0.66-0.94) |
| Sweden | Full group | 0.72 (0.68-0.77) | 0.69 (0.65-0.74) | 0.69 (0.65-0.74) |
|  | Male | 0.70 (0.63-0.76) | 0.68 (0.62-0.75) | 0.68 (0.62-0.74) |
|  | Female | 0.75 (0.68-0.82) | 0.70 (0.64-0.77) | 0.70 (0.64-0.77) |
|  | Young adults | 0.65 (0.58-0.73) | 0.63 (0.56-0.71) | 0.62 (0.55-0.70) |
|  | Middle-aged adults | 0.76 (0.70-0.82) | 0.73 (0.67-0.79) | 0.73 (0.67-0.79) |
|  | Older adults | 0.87 (0.52-1.42) | 0.89 (0.53-1.48) | 0.88 (0.52-1.47) |
| UK | Full group | 0.58 (0.36-0.92) | 0.64 (0.39-1.04) | 0.64 (0.38-1.05) |
|  | Male | 0.50 (0.25-0.96) | 0.60 (0.29-1.20) | 0.56 (0.26-1.16) |
|  | Female | 0.63 (0.32-1.23) | 0.68 (0.34-1.38) | 0.64 (0.31-1.32) |
|  | Young adults | 0.71 (0.34-1.48) | 0.76 (0.35-1.68) | 0.72 (0.32-1.61) |
|  | Middle-aged adults | 0.72 (0.36-1.38) | 0.71 (0.35-1.41) | 0.72 (0.34-1.50) |
| US | Full group | 0.44 (0.43-0.45) | 0.43 (0.42-0.44) | 0.43 (0.42-0.44) |
|  | Male | 0.46 (0.45-0.48) | 0.46 (0.45-0.48) | 0.46 (0.44-0.47) |
|  | Female | 0.43 (0.41-0.44) | 0.42 (0.40-0.43) | 0.41 (0.40-0.43) |
|  | Young adults | 0.47 (0.45-0.50) | 0.47 (0.45-0.49) | 0.46 (0.44-0.48) |
|  | Middle-aged adults | 0.44 (0.43-0.45) | 0.44 (0.43-0.45) | 0.44 (0.42-0.45) |
|  | Older adults | 0.34 (0.31-0.36) | 0.33 (0.31-0.36) | 0.33 (0.31-0.36) |
| Pooled analysis (including US results) | Full group | 0.63 (0.54-0.74) | 0.62 (0.53-0.72) | 0.62 (0.53-0.72) |
|  | Male | 0.61 (0.53-0.71) | 0.61 (0.53-0.70) | 0.60 (0.52-0.70) |
|  | Female | 0.64 (0.53-0.77) | 0.62 (0.52-0.74) | 0.62 (0.51-0.75) |
|  | Young adults | 0.58 (0.51-0.67) | 0.56 (0.50-0.63) | 0.56 (0.49-0.63) |
|  | Middle-aged adults | 0.66 (0.56-0.79) | 0.65 (0.55-0.77) | 0.65 (0.54-0.78) |
|  | Older adults | 0.64 (0.43-0.95) | 0.66 (0.44-0.98) | 0.66 (0.44-0.99) |
| Pooled analysis (excluding US results) | Full group | 0.68 (0.61-0.75) | 0.66 (0.60-0.73) | 0.66 (0.60-0.73) |
|  | Male | 0.66 (0.59-0.73) | 0.65 (0.58-0.71) | 0.64 (0.58-0.71) |
|  | Female | 0.70 (0.62-0.78) | 0.67 (0.59-0.75) | 0.67 (0.59-0.75) |
|  | Young adults | 0.61 (0.53-0.70) | 0.59 (0.53-0.65) | 0.58 (0.52-0.65) |
|  | Middle-aged adults | 0.72 (0.65-0.80) | 0.70 (0.63-0.77) | 0.70 (0.63-0.78) |
|  | Older adults | 0.73 (0.62-0.86) | 0.75 (0.64-0.88) | 0.75 (0.64-0.88) |

Models were adjusted for age, sex, and calendar year at the time of first dispensation of antihypertensive medication. In age-stratified analyses, age was not included as an adjustment variable.

Abbreviations: ADHD, attention-deficit/hyperactivity disorder; CI, confidence interval; OR, odds ratio; UK, United Kingdom; US, United States.

# Table 13. Association between ADHD medication and poor adherence to antihypertensive medication among individuals with ADHD at 2-year follow-up.

| **Country** | **Subgroups** | **Model 1**  **OR (95% CI)** | **Model 2**  **OR (95% CI)** | **Model 3**  **OR (95% CI)** |
| --- | --- | --- | --- | --- |
| Australia | Full group | 0.79 (0.71-0.87) | 0.77 (0.69-0.85) | 0.77 (0.69-0.85) |
|  | Male | 0.75 (0.66-0.86) | 0.74 (0.65-0.85) | 0.74 (0.65-0.85) |
|  | Female | 0.82 (0.70-0.96) | 0.80 (0.68-0.94) | 0.80 (0.68-0.95) |
|  | Young adults | 0.68 (0.57-0.82) | 0.63 (0.52-0.76) | 0.63 (0.52-0.76) |
|  | Middle-aged adults | 0.85 (0.75-0.97) | 0.84 (0.73-0.95) | 0.84 (0.74-0.96) |
|  | Older adults | 0.81 (0.52-1.27) | 0.80 (0.51-1.25) | 0.82 (0.52-1.29) |
| Denmark | Full group | 0.47 (0.43-0.50) | 0.45 (0.41-0.48) | 0.45 (0.41-0.48) |
|  | Male | 0.44 (0.39-0.50) | 0.43 (0.38-0.48) | 0.42 (0.38-0.48) |
|  | Female | 0.49 (0.44-0.55) | 0.46 (0.41-0.52) | 0.46 (0.41-0.52) |
|  | Young adults | 0.37 (0.32-0.43) | 0.38 (0.32-0.44) | 0.38 (0.32-0.44) |
|  | Middle-aged adults | 0.48 (0.43-0.53) | 0.47 (0.42-0.52) | 0.47 (0.42-0.52) |
|  | Older adults | 0.55 (0.37-0.81) | 0.60 (0.40-0.89) | 0.60 (0.40-0.90) |
| Netherlands | Full group | 0.68 (0.64-0.72) | 0.63 (0.59-0.66) | 0.63 (0.59-0.66) |
|  | Male | 0.67 (0.62-0.73) | 0.63 (0.58-0.68) | 0.63 (0.58-0.68) |
|  | Female | 0.68 (0.63-0.73) | 0.62 (0.58-0.67) | 0.62 (0.58-0.67) |
|  | Young adults | 0.58 (0.52-0.65) | 0.55 (0.49-0.62) | 0.55 (0.49-0.62) |
|  | Middle-aged adults | 0.71 (0.66-0.76) | 0.67 (0.63-0.72) | 0.67 (0.63-0.72) |
|  | Older adults | 0.61 (0.51-0.74) | 0.61 (0.51-0.74) | 0.62 (0.51-0.74) |
| Norway | Full group | 0.69 (0.60-0.78) | 0.68 (0.60-0.78) | 0.68 (0.59-0.78) |
|  | Male | 0.62 (0.51-0.75) | 0.62 (0.51-0.76) | 0.62 (0.51-0.76) |
|  | Female | 0.73 (0.61-0.88) | 0.74 (0.61-0.89) | 0.74 (0.61-0.89) |
|  | Young adults | 0.67 (0.54-0.82) | 0.62 (0.50-0.77) | 0.62 (0.50-0.77) |
|  | Middle-aged adults | 0.73 (0.61-0.86) | 0.72 (0.61-0.86) | 0.72 (0.61-0.86) |
| Sweden | Full group | 0.76 (0.71-0.80) | 0.72 (0.67-0.76) | 0.71 (0.67-0.76) |
|  | Male | 0.72 (0.66-0.78) | 0.70 (0.64-0.76) | 0.69 (0.64-0.75) |
|  | Female | 0.79 (0.73-0.87) | 0.74 (0.67-0.81) | 0.73 (0.67-0.80) |
|  | Young adults | 0.72 (0.65-0.81) | 0.69 (0.62-0.77) | 0.68 (0.61-0.77) |
|  | Middle-aged adults | 0.76 (0.71-0.82) | 0.72 (0.67-0.78) | 0.72 (0.67-0.78) |
|  | Older adults | 0.94 (0.59-1.46) | 0.97 (0.61-1.53) | 0.99 (0.62-1.56) |
| UK | Full group | 0.48 (0.30-0.75) | 0.50 (0.31-0.82) | 0.50 (0.30-0.82) |
|  | Male | 0.40 (0.20-0.76) | 0.47 (0.23-0.92) | 0.46 (0.22-0.94) |
|  | Female | 0.53 (0.27-1.01) | 0.56 (0.28-1.09) | 0.48 (0.23-0.98) |
|  | Young adults | 0.61 (0.29-1.27) | 0.67 (0.30-1.49) | 0.68 (0.30-1.55) |
|  | Middle-aged adults | 0.54 (0.28-1.03) | 0.51 (0.25-0.99) | 0.47 (0.22-0.94) |
| US | Full group | 0.40 (0.39-0.41) | 0.39 (0.38-0.40) | 0.39 (0.38-0.40) |
|  | Male | 0.41 (0.40-0.43) | 0.41 (0.40-0.42) | 0.40 (0.39-0.42) |
|  | Female | 0.39 (0.38-0.40) | 0.38 (0.37-0.39) | 0.37 (0.36-0.38) |
|  | Young adults | 0.44 (0.42-0.46) | 0.43 (0.41-0.45) | 0.42 (0.40-0.44) |
|  | Middle-aged adults | 0.40 (0.39-0.41) | 0.39 (0.38-0.40) | 0.39 (0.38-0.40) |
|  | Older adults | 0.30 (0.28-0.32) | 0.30 (0.28-0.32) | 0.30 (0.28-0.32) |
| Pooled analysis (including US results) | Full group | 0.59 (0.48-0.72) | 0.57 (0.47-0.70) | 0.57 (0.47-0.70) |
|  | Male | 0.56 (0.46-0.68) | 0.55 (0.46-0.66) | 0.55 (0.46-0.66) |
|  | Female | 0.62 (0.49-0.77) | 0.59 (0.47-0.74) | 0.59 (0.47-0.74) |
|  | Young adults | 0.56 (0.46-0.68) | 0.53 (0.45-0.63) | 0.53 (0.45-0.63) |
|  | Middle-aged adults | 0.62 (0.49-0.77) | 0.60 (0.48-0.74) | 0.60 (0.48-0.74) |
|  | Older adults | 0.58 (0.39-0.86) | 0.59 (0.39-0.89) | 0.59 (0.39-0.90) |
| Pooled analysis (excluding US results) | Full group | 0.64 (0.54-0.76) | 0.62 (0.52-0.73) | 0.62 (0.52-0.73) |
|  | Male | 0.60 (0.51-0.71) | 0.59 (0.50-0.70) | 0.59 (0.49-0.70) |
|  | Female | 0.68 (0.57-0.81) | 0.65 (0.54-0.78) | 0.65 (0.54-0.78) |
|  | Young adults | 0.58 (0.47-0.72) | 0.56 (0.46-0.67) | 0.56 (0.46-0.67) |
|  | Middle-aged adults | 0.67 (0.56-0.81) | 0.65 (0.55-0.78) | 0.65 (0.54-0.78) |
|  | Older adults | 0.67 (0.55-0.82) | 0.68 (0.56-0.84) | 0.69 (0.56-0.85) |

Models were adjusted for age, sex, and calendar year at the time of first dispensation of antihypertensive medication. In age-stratified analyses, age was not included as an adjustment variable.

Abbreviations: ADHD, attention-deficit/hyperactivity disorder; CI, confidence interval; OR, odds ratio; UK, United Kingdom; US, United States.

# Table 14. Association between ADHD medication and poor adherence to antihypertensive medication among individuals with ADHD at 5-year follow-up

| **Country** | **Subgroups** | **Model 1**  **OR (95% CI)** | **Model 2**  **OR (95% CI)** | **Model 3**  **OR (95% CI)** |
| --- | --- | --- | --- | --- |
| Australia | Full group | 0.79 (0.71-0.87) | 0.76 (0.68-0.84) | 0.76 (0.68-0.84) |
|  | Male | 0.74 (0.65-0.84) | 0.72 (0.63-0.82) | 0.72 (0.63-0.82) |
|  | Female | 0.84 (0.72-0.98) | 0.82 (0.69-0.96) | 0.82 (0.70-0.97) |
|  | Young adults | 0.74 (0.62-0.89) | 0.67 (0.55-0.81) | 0.67 (0.55-0.81) |
|  | Middle-aged adults | 0.83 (0.73-0.94) | 0.80 (0.70-0.91) | 0.80 (0.71-0.91) |
|  | Older adults | 0.72 (0.46-1.12) | 0.70 (0.45-1.09) | 0.72 (0.45-1.13) |
| Denmark | Full group | 0.40 (0.37-0.43) | 0.37 (0.34-0.40) | 0.37 (0.34-0.40) |
|  | Male | 0.35 (0.31-0.39) | 0.33 (0.29-0.37) | 0.33 (0.29-0.37) |
|  | Female | 0.45 (0.40-0.50) | 0.41 (0.37-0.47) | 0.41 (0.37-0.47) |
|  | Young adults | 0.32 (0.27-0.38) | 0.32 (0.27-0.38) | 0.32 (0.27-0.38) |
|  | Middle-aged adults | 0.39 (0.35-0.43) | 0.38 (0.34-0.42) | 0.37 (0.34-0.41) |
|  | Older adults | 0.50 (0.34-0.73) | 0.54 (0.36-0.79) | 0.54 (0.36-0.79) |
| Netherlands | Full group | 0.68 (0.65-0.72) | 0.61 (0.58-0.65) | 0.61 (0.58-0.64) |
|  | Male | 0.69 (0.64-0.74) | 0.62 (0.58-0.67) | 0.62 (0.57-0.67) |
|  | Female | 0.68 (0.63-0.73) | 0.60 (0.56-0.65) | 0.60 (0.56-0.65) |
|  | Young adults | 0.59 (0.53-0.66) | 0.53 (0.47-0.59) | 0.52 (0.46-0.59) |
|  | Middle-aged adults | 0.70 (0.66-0.75) | 0.65 (0.61-0.70) | 0.65 (0.61-0.70) |
|  | Older adults | 0.63 (0.53-0.75) | 0.63 (0.52-0.75) | 0.63 (0.53-0.76) |
| Norway | Full group | 0.68 (0.60-0.78) | 0.68 (0.59-0.78) | 0.67 (0.59-0.77) |
|  | Male | 0.62 (0.51-0.75) | 0.62 (0.51-0.76) | 0.62 (0.51-0.75) |
|  | Female | 0.73 (0.61-0.87) | 0.73 (0.61-0.88) | 0.73 (0.61-0.88) |
|  | Young adults | 0.64 (0.52-0.78) | 0.58 (0.47-0.72) | 0.58 (0.47-0.72) |
|  | Middle-aged adults | 0.74 (0.63-0.88) | 0.74 (0.62-0.88) | 0.73 (0.62-0.87) |
| Sweden | Full group | 0.75 (0.71-0.80) | 0.69 (0.65-0.74) | 0.69 (0.65-0.73) |
|  | Male | 0.70 (0.64-0.76) | 0.66 (0.60-0.71) | 0.65 (0.60-0.70) |
|  | Female | 0.82 (0.75-0.89) | 0.74 (0.67-0.81) | 0.73 (0.67-0.80) |
|  | Young adults | 0.72 (0.65-0.80) | 0.65 (0.58-0.73) | 0.65 (0.58-0.73) |
|  | Middle-aged adults | 0.75 (0.70-0.81) | 0.70 (0.65-0.75) | 0.70 (0.65-0.75) |
|  | Older adults | 1.04 (0.68-1.59) | 1.07 (0.69-1.64) | 1.07 (0.69-1.64) |
| UK | Full group | 0.40 (0.26-0.63) | 0.42 (0.26-0.67) | 0.42 (0.26-0.67) |
|  | Male | 0.32 (0.17-0.59) | 0.36 (0.18-0.69) | 0.34 (0.17-0.68) |
|  | Female | 0.50 (0.26-0.95) | 0.51 (0.26-0.99) | 0.46 (0.22-0.92) |
|  | Young adults | 0.51 (0.24-1.07) | 0.57 (0.26-1.27) | 0.55 (0.24-1.24) |
|  | Middle-aged adults | 0.41 (0.22-0.77) | 0.38 (0.20-0.73) | 0.35 (0.17-0.69) |
| US | Full group | 0.40 (0.39-0.41) | 0.38 (0.38-0.39) | 0.38 (0.37-0.39) |
|  | Male | 0.40 (0.39-0.41) | 0.40 (0.38-0.41) | 0.39 (0.38-0.41) |
|  | Female | 0.39 (0.38-0.40) | 0.38 (0.36-0.39) | 0.37 (0.36-0.38) |
|  | Young adults | 0.45 (0.43-0.48) | 0.45 (0.42-0.47) | 0.44 (0.42-0.46) |
|  | Middle-aged adults | 0.39 (0.38-0.40) | 0.38 (0.37-0.39) | 0.38 (0.37-0.39) |
|  | Older adults | 0.30 (0.28-0.32) | 0.30 (0.28-0.32) | 0.30 (0.28-0.32) |
| Pooled analysis (including US results) | Full group | 0.57 (0.45-0.72) | 0.54 (0.43-0.68) | 0.54 (0.43-0.68) |
|  | Male | 0.53 (0.41-0.67) | 0.51 (0.41-0.65) | 0.51 (0.40-0.65) |
|  | Female | 0.61 (0.48-0.78) | 0.58 (0.45-0.74) | 0.57 (0.45-0.73) |
|  | Young adults | 0.55 (0.43-0.69) | 0.51 (0.42-0.62) | 0.51 (0.42-0.62) |
|  | Middle-aged adults | 0.58 (0.45-0.75) | 0.56 (0.43-0.72) | 0.55 (0.43-0.72) |
|  | Older adults | 0.56 (0.38-0.84) | 0.57 (0.38-0.86) | 0.57 (0.38-0.87) |
| Pooled analysis (excluding US results) | Full group | 0.61 (0.48-0.76) | 0.58 (0.46-0.73) | 0.58 (0.46-0.72) |
|  | Male | 0.56 (0.43-0.72) | 0.54 (0.42-0.70) | 0.54 (0.41-0.69) |
|  | Female | 0.67 (0.54-0.82) | 0.63 (0.50-0.78) | 0.63 (0.50-0.78) |
|  | Young adults | 0.57 (0.44-0.74) | 0.53 (0.42-0.67) | 0.52 (0.42-0.66) |
|  | Middle-aged adults | 0.63 (0.49-0.81) | 0.60 (0.47-0.77) | 0.60 (0.46-0.77) |
|  | Older adults | 0.66 (0.53-0.83) | 0.67 (0.55-0.83) | 0.67 (0.55-0.83) |

Models were adjusted for age, sex, and calendar year at the time of first dispensation of antihypertensive medication. In age-stratified analyses, age was not included as an adjustment variable.

Abbreviations: ADHD, attention-deficit/hyperactivity disorder; CI, confidence interval; OR, odds ratio; UK, United Kingdom; US, United States.

# Table 15. Sensitivity analysis: association between ADHD and first discontinuation of antihypertensive medication treatment after using ADHD as a lifetime exposure

| **Country** | **Subgroups** | **Model 1**  **HR (95% CI)** | **Model 2**  **HR (95% CI)** | **Model 3**  **HR (95% CI)** |
| --- | --- | --- | --- | --- |
| Australia | Full group | 1.26 (1.22-1.29) | 1.12 (1.09-1.15) | 1.08 (1.05-1.11) |
|  | Male | 1.15 (1.11-1.20) | 1.10 (1.06-1.14) | 1.07 (1.03-1.11) |
|  | Female | 1.39 (1.34-1.44) | 1.15 (1.11-1.20) | 1.11 (1.07-1.15) |
|  | Young adults | 0.99 (0.95-1.03) | 0.97 (0.93-1.01) | 0.97 (0.93-1.01) |
|  | Middle-aged adults | 1.15 (1.11-1.19) | 1.08 (1.04-1.12) | 1.03 (1.00-1.07) |
|  | Older adults | 0.95 (0.82-1.10) | 1.04 (0.90-1.21) | 1.01 (0.87-1.16) |
| Denmark | Full group | 1.38 (1.36-1.41) | 1.20 (1.18-1.23) | 1.16 (1.14-1.19) |
|  | Male | 1.38 (1.34-1.42) | 1.28 (1.24-1.31) | 1.23 (1.19-1.26) |
|  | Female | 1.39 (1.35-1.43) | 1.15 (1.12-1.19) | 1.13 (1.10-1.16) |
|  | Young adults | 0.98 (0.95-1.02) | 0.98 (0.95-1.02) | 1.00 (0.96-1.04) |
|  | Middle-aged adults | 1.28 (1.25-1.32) | 1.14 (1.11-1.17) | 1.10 (1.07-1.13) |
|  | Older adults | 1.24 (1.15-1.33) | 1.31 (1.22-1.40) | 1.30 (1.21-1.40) |
| Netherlands | Full group | 1.42 (1.41-1.44) | 1.29 (1.28-1.31) | 1.25 (1.23-1.26) |
|  | Male | 1.39 (1.37-1.42) | 1.33 (1.31-1.36) | 1.28 (1.26-1.30) |
|  | Female | 1.47 (1.44-1.50) | 1.27 (1.25-1.30) | 1.23 (1.21-1.25) |
|  | Young adults | 1.00 (0.97-1.02) | 1.02 (0.99-1.05) | 1.03 (1.00-1.06) |
|  | Middle-aged adults | 1.35 (1.33-1.37) | 1.24 (1.22-1.26) | 1.18 (1.16-1.20) |
|  | Older adults | 1.26 (1.22-1.31) | 1.27 (1.22-1.31) | 1.24 (1.20-1.29) |
| Norway | Full group | 1.19 (1.15-1.23) | 1.11 (1.07-1.15) | 1.08 (1.04-1.12) |
|  | Male | 1.19 (1.13-1.26) | 1.12 (1.06-1.18) | 1.07 (1.02-1.13) |
|  | Female | 1.19 (1.14-1.24) | 1.11 (1.06-1.16) | 1.09 (1.04-1.14) |
|  | Young adults | 1.04 (0.98-1.09) | 1.05 (1.00-1.10) | 1.06 (1.00-1.12) |
|  | Middle-aged adults | 1.19 (1.14-1.24) | 1.16 (1.11-1.21) | 1.10 (1.05-1.15) |
| Sweden | Full group | 0.98 (0.97-1.00) | 1.09 (1.08-1.11) | 1.05 (1.03-1.06) |
|  | Male | 0.96 (0.94-0.98) | 1.11 (1.09-1.14) | 1.07 (1.04-1.09) |
|  | Female | 1.01 (0.99-1.04) | 1.09 (1.06-1.11) | 1.04 (1.02-1.07) |
|  | Young adults | 0.92 (0.89-0.94) | 0.96 (0.93-0.98) | 0.98 (0.94-1.01) |
|  | Middle-aged adults | 1.04 (1.02-1.06) | 1.03 (1.01-1.05) | 0.99 (0.97-1.01) |
|  | Older adults | 1.00 (0.91-1.10) | 1.04 (0.95-1.15) | 1.00 (0.91-1.10) |
| UK | Full group | 1.63 (1.46-1.82) | 1.37 (1.22-1.53) | 1.16 (1.04-1.30) |
|  | Male | 1.69 (1.46-1.96) | 1.48 (1.28-1.72) | 1.28 (1.10-1.49) |
|  | Female | 1.65 (1.40-1.94) | 1.32 (1.12-1.55) | 1.10 (0.93-1.29) |
|  | Young adults | 1.09 (0.92-1.29) | 1.16 (0.98-1.38) | 1.15 (0.97-1.37) |
|  | Middle-aged adults | 1.38 (1.18-1.62) | 1.17 (1.00-1.37) | 0.96 (0.82-1.12) |
|  | Older adults | 1.33 (0.88-2.00) | 1.42 (0.94-2.13) | 1.29 (0.86-1.94) |
| US | Full group | 0.93 (0.92-0.93) | 0.81 (0.81-0.82) | 0.82 (0.81-0.82) |
|  | Male | 0.89 (0.89-0.90) | 0.81 (0.80-0.81) | 0.81 (0.80-0.81) |
|  | Female | 0.96 (0.95-0.96) | 0.82 (0.81-0.82) | 0.82 (0.82-0.83) |
|  | Young adults | 0.82 (0.81-0.82) | 0.82 (0.81-0.82) | 0.83 (0.82-0.83) |
|  | Middle-aged adults | 0.83 (0.82-0.83) | 0.83 (0.83-0.84) | 0.84 (0.83-0.84) |
|  | Older adults | 0.83 (0.82-0.84) | 0.82 (0.81-0.83) | 0.82 (0.81-0.83) |

Model 1 was the crude model; Model 2 adjusted for age, sex, and calendar year at the time of first dispensation of antihypertensive medication; Model 3 additionally adjusted for other psychiatric comorbidities. In age-stratified analyses, age was not included as an adjustment variable.

Abbreviations: ADHD, attention-deficit/hyperactivity disorder; CI, confidence interval; HR, hazard ratio; UK, United Kingdom; US, United States.

# Table 16. Sensitivity analysis: association between ADHD and poor adherence to antihypertensive medication at 1-year follow-up after using ADHD as a lifetime exposure

| **Country** | **Subgroups** | **Model 1**  **OR (95% CI)** | **Model 2**  **OR (95% CI)** | **Model 3**  **OR (95% CI)** |
| --- | --- | --- | --- | --- |
| Australia | Full group | 1.51 (1.41-1.60) | 1.20 (1.13-1.28) | 1.14 (1.07-1.21) |
|  | Male | 1.35 (1.25-1.47) | 1.16 (1.07-1.26) | 1.12 (1.03-1.22) |
|  | Female | 1.83 (1.66-2.03) | 1.33 (1.20-1.47) | 1.23 (1.11-1.37) |
|  | Young adults | 1.05 (0.93-1.18) | 0.95 (0.84-1.08) | 0.96 (0.85-1.09) |
|  | Middle-aged adults | 1.23 (1.14-1.33) | 1.07 (0.99-1.16) | 1.01 (0.94-1.10) |
|  | Older adults | 0.94 (0.73-1.22) | 1.03 (0.80-1.33) | 0.97 (0.75-1.26) |
| Denmark | Full group | 1.78 (1.69-1.86) | 1.47 (1.40-1.54) | 1.34 (1.28-1.41) |
|  | Male | 1.91 (1.79-2.05) | 1.84 (1.72-1.97) | 1.64 (1.53-1.76) |
|  | Female | 1.69 (1.58-1.81) | 1.25 (1.16-1.33) | 1.16 (1.08-1.25) |
|  | Young adults | 0.87 (0.79-0.96) | 0.87 (0.78-0.96) | 0.92 (0.83-1.02) |
|  | Middle-aged adults | 1.54 (1.45-1.63) | 1.27 (1.19-1.34) | 1.17 (1.10-1.25) |
|  | Older adults | 2.68 (2.22-3.24) | 2.71 (2.24-3.28) | 2.54 (2.10-3.07) |
| Netherlands | Full group | 1.58 (1.54-1.63) | 1.38 (1.34-1.42) | 1.32 (1.28-1.36) |
|  | Male | 1.60 (1.54-1.67) | 1.47 (1.41-1.53) | 1.39 (1.33-1.45) |
|  | Female | 1.63 (1.57-1.69) | 1.34 (1.29-1.39) | 1.28 (1.23-1.33) |
|  | Young adults | 0.99 (0.93-1.05) | 1.00 (0.94-1.06) | 1.02 (0.96-1.09) |
|  | Middle-aged adults | 1.48 (1.43-1.53) | 1.28 (1.23-1.32) | 1.19 (1.15-1.24) |
|  | Older adults | 1.41 (1.31-1.52) | 1.41 (1.31-1.52) | 1.37 (1.27-1.48) |
| Norway | Full group | 1.54 (1.42-1.66) | 1.29 (1.19-1.40) | 1.14 (1.05-1.24) |
|  | Male | 1.60 (1.43-1.79) | 1.31 (1.17-1.47) | 1.10 (0.97-1.23) |
|  | Female | 1.55 (1.39-1.74) | 1.28 (1.14-1.44) | 1.19 (1.06-1.34) |
|  | Young adults | 1.08 (0.96-1.23) | 1.07 (0.94-1.22) | 1.02 (0.89-1.16) |
|  | Middle-aged adults | 1.57 (1.42-1.74) | 1.47 (1.33-1.63) | 1.25 (1.12-1.39) |
| Sweden | Full group | 2.07 (2.00-2.15) | 1.55 (1.49-1.60) | 1.13 (1.09-1.17) |
|  | Male | 1.89 (1.80-1.98) | 1.74 (1.65-1.82) | 1.27 (1.21-1.34) |
|  | Female | 2.43 (2.30-2.56) | 1.50 (1.42-1.58) | 1.10 (1.04-1.16) |
|  | Young adults | 0.80 (0.75-0.85) | 0.86 (0.80-0.93) | 0.85 (0.79-0.92) |
|  | Middle-aged adults | 1.77 (1.70-1.85) | 1.43 (1.37-1.50) | 1.01 (0.96-1.06) |
|  | Older adults | 1.80 (1.45-2.23) | 1.95 (1.57-2.42) | 1.53 (1.23-1.90) |
| UK | Full group | 1.62 (1.27-2.07) | 1.28 (1.00-1.65) | 1.19 (0.92-1.52) |
|  | Male | 1.82 (1.31-2.52) | 1.52 (1.09-2.11) | 1.40 (1.01-1.96) |
|  | Female | 1.53 (1.06-2.23) | 1.13 (0.78-1.66) | 1.03 (0.71-1.51) |
|  | Young adults | 0.78 (0.53-1.15) | 0.84 (0.57-1.26) | 0.90 (0.61-1.35) |
|  | Middle-aged adults | 1.34 (0.93-1.91) | 1.09 (0.75-1.56) | 0.96 (0.67-1.38) |
|  | Older adults | 0.80 (0.30-1.91) | 0.88 (0.33-2.12) | 0.82 (0.31-2.00) |

Models were adjusted for age, sex, and calendar year at the time of first dispensation of antihypertensive medication. In age-stratified analyses, age was not included as an adjustment variable.

Abbreviations: ADHD, attention-deficit/hyperactivity disorder; CI, confidence interval; OR, odds ratio; UK, United Kingdom; US, United States.

# Table 17. Sensitivity analysis: association between ADHD and poor adherence to antihypertensive medication at 2-year follow-up after using ADHD as a lifetime exposure

| **Country** | **Subgroups** | **Model 1**  **OR (95% CI)** | **Model 2**  **OR (95% CI)** | **Model 3**  **OR (95% CI)** |
| --- | --- | --- | --- | --- |
| Australia | Full group | 1.72 (1.60-1.84) | 1.21 (1.13-1.30) | 1.14 (1.07-1.23) |
|  | Male | 1.51 (1.39-1.65) | 1.15 (1.05-1.26) | 1.11 (1.01-1.21) |
|  | Female | 2.17 (1.94-2.44) | 1.39 (1.24-1.57) | 1.29 (1.15-1.45) |
|  | Young adults | 1.13 (0.98-1.30) | 0.92 (0.80-1.07) | 0.94 (0.82-1.09) |
|  | Middle-aged adults | 1.42 (1.31-1.55) | 1.12 (1.02-1.22) | 1.05 (0.97-1.15) |
|  | Older adults | 0.90 (0.69-1.17) | 0.89 (0.69-1.17) | 0.83 (0.64-1.09) |
| Denmark | Full group | 1.97 (1.88-2.07) | 1.49 (1.41-1.56) | 1.34 (1.27-1.41) |
|  | Male | 2.13 (1.99-2.29) | 1.84 (1.71-1.97) | 1.61 (1.50-1.73) |
|  | Female | 1.85 (1.73-1.99) | 1.25 (1.17-1.35) | 1.16 (1.08-1.25) |
|  | Young adults | 0.89 (0.80-0.99) | 0.83 (0.75-0.93) | 0.87 (0.78-0.98) |
|  | Middle-aged adults | 1.72 (1.62-1.83) | 1.31 (1.23-1.39) | 1.19 (1.12-1.27) |
|  | Older adults | 2.84 (2.33-3.49) | 2.86 (2.34-3.52) | 2.66 (2.17-3.28) |
| Netherlands | Full group | 1.58 (1.54-1.62) | 1.40 (1.36-1.44) | 1.36 (1.32-1.40) |
|  | Male | 1.68 (1.62-1.74) | 1.49 (1.43-1.55) | 1.43 (1.37-1.48) |
|  | Female | 1.56 (1.50-1.62) | 1.33 (1.28-1.39) | 1.30 (1.25-1.35) |
|  | Young adults | 1.06 (1.00-1.14) | 1.02 (0.95-1.10) | 1.06 (0.98-1.14) |
|  | Middle-aged adults | 1.56 (1.51-1.61) | 1.27 (1.23-1.32) | 1.20 (1.16-1.25) |
|  | Older adults | 1.35 (1.26-1.46) | 1.35 (1.25-1.45) | 1.34 (1.24-1.44) |
| Norway | Full group | 1.54 (1.42-1.68) | 1.22 (1.12-1.34) | 1.08 (0.99-1.18) |
|  | Male | 1.61 (1.43-1.81) | 1.27 (1.12-1.43) | 1.08 (0.95-1.22) |
|  | Female | 1.54 (1.36-1.74) | 1.19 (1.05-1.36) | 1.10 (0.96-1.25) |
|  | Young adults | 1.08 (0.94-1.24) | 1.02 (0.88-1.17) | 0.98 (0.84-1.13) |
|  | Middle-aged adults | 1.55 (1.39-1.73) | 1.38 (1.24-1.55) | 1.17 (1.04-1.31) |
| Sweden | Full group | 2.39 (2.31-2.48) | 1.61 (1.55-1.67) | 1.20 (1.16-1.25) |
|  | Male | 2.21 (2.10-2.32) | 1.77 (1.68-1.86) | 1.35 (1.28-1.42) |
|  | Female | 2.75 (2.61-2.91) | 1.56 (1.48-1.65) | 1.16 (1.09-1.23) |
|  | Young adults | 0.92 (0.86-0.99) | 0.93 (0.86-1.01) | 0.96 (0.88-1.05) |
|  | Middle-aged adults | 2.00 (1.91-2.09) | 1.45 (1.39-1.52) | 1.05 (1.00-1.10) |
|  | Older adults | 1.77 (1.43-2.18) | 1.66 (1.34-2.05) | 1.34 (1.08-1.66) |
| UK | Full group | 1.82 (1.42-2.35) | 1.36 (1.06-1.77) | 1.26 (0.97-1.64) |
|  | Male | 2.05 (1.47-2.88) | 1.65 (1.18-2.32) | 1.53 (1.09-2.16) |
|  | Female | 1.70 (1.16-2.52) | 1.17 (0.80-1.76) | 1.06 (0.72-1.60) |
|  | Young adults | 1.07 (0.69-1.70) | 1.08 (0.69-1.74) | 1.14 (0.73-1.84) |
|  | Middle-aged adults | 1.35 (0.95-1.93) | 1.02 (0.71-1.47) | 0.92 (0.64-1.32) |
|  | Older adults | 0.68 (0.27-1.63) | 0.73 (0.29-1.76) | 0.68 (0.26-1.64) |

Models were adjusted for age, sex, and calendar year at the time of first dispensation of antihypertensive medication. In age-stratified analyses, age was not included as an adjustment variable.

Abbreviations: ADHD, attention-deficit/hyperactivity disorder; CI, confidence interval; OR, odds ratio; UK, United Kingdom; US, United States.

# Table 18. Sensitivity analysis: association between ADHD and poor adherence to antihypertensive medication at 5-year follow-up after using ADHD as a lifetime exposure

| **Country** | **Subgroups** | **Model 1**  **OR (95% CI)** | **Model 2**  **OR (95% CI)** | **Model 3**  **OR (95% CI)** |
| --- | --- | --- | --- | --- |
| Australia | Full group | 2.20 (2.02-2.42) | 1.24 (1.13-1.36) | 1.15 (1.05-1.27) |
|  | Male | 1.92 (1.72-2.15) | 1.16 (1.03-1.31) | 1.10 (0.98-1.24) |
|  | Female | 2.89 (2.48-3.40) | 1.48 (1.26-1.75) | 1.34 (1.14-1.59) |
|  | Young adults | 1.59 (1.30-1.97) | 1.00 (0.81-1.25) | 1.01 (0.81-1.26) |
|  | Middle-aged adults | 1.78 (1.60-1.98) | 1.09 (0.98-1.23) | 1.02 (0.91-1.15) |
|  | Older adults | 1.28 (0.94-1.78) | 1.00 (0.72-1.41) | 0.91 (0.65-1.29) |
| Denmark | Full group | 2.29 (2.16-2.43) | 1.54 (1.45-1.63) | 1.36 (1.28-1.45) |
|  | Male | 2.38 (2.20-2.58) | 1.83 (1.69-2.00) | 1.59 (1.46-1.73) |
|  | Female | 2.23 (2.05-2.43) | 1.35 (1.23-1.47) | 1.22 (1.12-1.34) |
|  | Young adults | 1.07 (0.94-1.22) | 0.87 (0.76-1.00) | 0.88 (0.76-1.02) |
|  | Middle-aged adults | 2.03 (1.90-2.18) | 1.31 (1.22-1.41) | 1.18 (1.10-1.27) |
|  | Older adults | 2.95 (2.34-3.78) | 2.96 (2.32-3.82) | 2.75 (2.15-3.55) |
| Netherlands | Full group | 1.73 (1.68-1.78) | 1.41 (1.37-1.46) | 1.42 (1.37-1.46) |
|  | Male | 1.88 (1.81-1.96) | 1.51 (1.45-1.58) | 1.49 (1.43-1.56) |
|  | Female | 1.65 (1.58-1.72) | 1.31 (1.26-1.38) | 1.32 (1.26-1.38) |
|  | Young adults | 1.27 (1.16-1.38) | 1.04 (0.95-1.14) | 1.12 (1.02-1.24) |
|  | Middle-aged adults | 1.78 (1.72-1.84) | 1.28 (1.23-1.32) | 1.25 (1.20-1.30) |
|  | Older adults | 1.25 (1.16-1.36) | 1.21 (1.11-1.32) | 1.24 (1.14-1.35) |
| Norway | Full group | 1.58 (1.43-1.76) | 1.12 (1.00-1.25) | 1.01 (0.90-1.14) |
|  | Male | 1.63 (1.42-1.88) | 1.17 (1.01-1.37) | 1.02 (0.87-1.20) |
|  | Female | 1.58 (1.35-1.85) | 1.07 (0.91-1.28) | 1.03 (0.87-1.22) |
|  | Young adults | 1.27 (1.06-1.53) | 1.04 (0.86-1.27) | 1.00 (0.82-1.23) |
|  | Middle-aged adults | 1.48 (1.31-1.69) | 1.19 (1.04-1.37) | 1.05 (0.91-1.21) |
| Sweden | Full group | 2.59 (2.48-2.70) | 1.51 (1.44-1.57) | 1.21 (1.15-1.27) |
|  | Male | 2.39 (2.27-2.53) | 1.65 (1.56-1.75) | 1.36 (1.28-1.45) |
|  | Female | 2.94 (2.76-3.14) | 1.46 (1.36-1.56) | 1.15 (1.07-1.23) |
|  | Young adults | 1.00 (0.91-1.10) | 0.85 (0.77-0.94) | 0.89 (0.80-1.00) |
|  | Middle-aged adults | 2.19 (2.09-2.30) | 1.31 (1.25-1.38) | 1.03 (0.97-1.08) |
|  | Older adults | 2.24 (1.78-2.85) | 1.57 (1.23-2.01) | 1.46 (1.14-1.88) |
| UK | Full group | 2.08 (1.56-2.81) | 1.47 (1.09-2.00) | 1.39 (1.03-1.90) |
|  | Male | 2.98 (2.00-4.59) | 2.23 (1.48-3.46) | 2.13 (1.42-3.32) |
|  | Female | 1.41 (0.94-2.19) | 0.93 (0.61-1.46) | 0.86 (0.56-1.35) |
|  | Young adults | 1.72 (0.94-3.51) | 1.44 (0.78-2.97) | 1.52 (0.82-3.15) |
|  | Middle-aged adults | 1.47 (1.01-2.17) | 0.98 (0.66-1.46) | 0.91 (0.62-1.37) |
|  | Older adults | 0.89 (0.38-2.25) | 0.94 (0.38-2.44) | 0.88 (0.36-2.32) |

Models were adjusted for age, sex, and calendar year at the time of first dispensation of antihypertensive medication. In age-stratified analyses, age was not included as an adjustment variable.

Abbreviations: ADHD, attention-deficit/hyperactivity disorder; CI, confidence interval; OR, odds ratio; UK, United Kingdom; US, United States.

# Table 19. Sensitivity analysis: association between ADHD and first discontinuation of antihypertensive medication treatment by using the country-specific gap to define discontinuation

| **Country** | **Subgroups** | **Model 1**  **HR (95% CI)** | **Model 2**  **HR (95% CI)** | **Model 3**  **HR (95% CI)** |
| --- | --- | --- | --- | --- |
| Australia | Full group | 1.19 (1.15-1.24) | 1.11 (1.07-1.15) | 1.06 (1.02-1.10) |
|  | Male | 1.15 (1.10-1.21) | 1.12 (1.07-1.18) | 1.09 (1.03-1.14) |
|  | Female | 1.28 (1.21-1.34) | 1.12 (1.06-1.18) | 1.06 (1.01-1.12) |
|  | Young adults | 0.95 (0.90-1.00) | 0.96 (0.91-1.01) | 0.96 (0.91-1.02) |
|  | Middle-aged adults | 1.10 (1.05-1.15) | 1.08 (1.03-1.13) | 1.02 (0.97-1.07) |
|  | Older adults | 0.91 (0.77-1.08) | 1.03 (0.87-1.22) | 0.98 (0.83-1.17) |
| Denmark | Full group | 1.42 (1.38-1.45) | 1.15 (1.13-1.18) | 1.11 (1.08-1.13) |
|  | Male | 1.47 (1.42-1.52) | 1.28 (1.23-1.32) | 1.21 (1.17-1.26) |
|  | Female | 1.37 (1.33-1.42) | 1.07 (1.04-1.11) | 1.04 (1.00-1.07) |
|  | Young adults | 0.95 (0.90-0.99) | 0.94 (0.89-0.98) | 0.95 (0.91-1.00) |
|  | Middle-aged adults | 1.32 (1.28-1.36) | 1.11 (1.08-1.15) | 1.06 (1.03-1.10) |
|  | Older adults | 1.23 (1.09-1.38) | 1.20 (1.07-1.35) | 1.20 (1.06-1.34) |
| UK | Full group | 1.76 (1.55-2.00) | 1.43 (1.26-1.63) | 1.20 (1.06-1.37) |
|  | Male | 1.94 (1.63-2.31) | 1.65 (1.39-1.97) | 1.43 (1.20-1.70) |
|  | Female | 1.64 (1.35-1.98) | 1.31 (1.08-1.59) | 1.07 (0.88-1.30) |
|  | Young adults | 1.09 (0.90-1.32) | 1.17 (0.96-1.41) | 1.14 (0.94-1.38) |
|  | Middle-aged adults | 1.55 (1.28-1.87) | 1.29 (1.07-1.55) | 1.02 (0.85-1.24) |
|  | Older adults | 1.11 (0.68-1.80) | 1.18 (0.73-1.93) | 1.06 (0.65-1.73) |

Model 1 was the crude model; Model 2 adjusted for age, sex, and calendar year at the time of first dispensation of antihypertensive medication; Model 3 additionally adjusted for other psychiatric comorbidities. In age-stratified analyses, age was not included as an adjustment variable.

Abbreviations: ADHD, attention-deficit/hyperactivity disorder; CI, confidence interval; HR, hazard ratio; UK, United Kingdom; US, United States.

# Table 20. Sensitivity analysis: association between ADHD and poor adherence to antihypertensive medication by using the country-specific gap to define discontinuation

| **Year** | **Country** | **Subgroups** | **Model 1**  **OR (95% CI)** | **Model 2**  **OR (95% CI)** | **Model 3**  **OR (95% CI)** |
| --- | --- | --- | --- | --- | --- |
| 1-year | Australia | Full group | 1.57 (1.47-1.67) | 1.18 (1.11-1.26) | 1.12 (1.05-1.20) |
|  |  | Male | 1.39 (1.28-1.51) | 1.13 (1.04-1.23) | 1.09 (1.01-1.19) |
|  |  | Female | 1.95 (1.76-2.17) | 1.34 (1.21-1.49) | 1.24 (1.12-1.39) |
|  |  | Young adults | 0.99 (0.88-1.13) | 0.90 (0.79-1.03) | 0.92 (0.80-1.05) |
|  |  | Middle-aged adults | 1.25 (1.15-1.35) | 1.07 (0.99-1.17) | 1.02 (0.94-1.10) |
|  |  | Older adults | 0.94 (0.73-1.21) | 1.03 (0.79-1.33) | 0.97 (0.75-1.26) |
|  | Denmark | Full group | 1.33 (1.17-1.53) | 1.03 (0.90-1.18) | 1.15 (1.01-1.32) |
|  |  | Male | 1.34 (1.13-1.61) | 1.12 (0.94-1.35) | 1.23 (1.03-1.49) |
|  |  | Female | 1.34 (1.11-1.65) | 0.95 (0.78-1.16) | 1.08 (0.89-1.34) |
|  |  | Young adults | 0.72 (0.56-0.94) | 0.72 (0.56-0.95) | 0.84 (0.64-1.11) |
|  |  | Middle-aged adults | 1.19 (1.02-1.41) | 1.06 (0.90-1.25) | 1.15 (0.98-1.36) |
|  |  | Older adults | 3.00 (1.60-6.59) | 2.81 (1.50-6.19) | 2.87 (1.53-6.32) |
|  | UK | Full group | 1.83 (1.40-2.44) | 1.36 (1.03-1.81) | 1.29 (0.98-1.71) |
|  |  | Male | 1.86 (1.31-2.70) | 1.47 (1.03-2.14) | 1.41 (0.99-2.06) |
|  |  | Female | 1.88 (1.24-2.95) | 1.32 (0.86-2.08) | 1.21 (0.79-1.91) |
|  |  | Young adults | 0.94 (0.59-1.59) | 0.97 (0.60-1.66) | 1.06 (0.66-1.82) |
|  |  | Middle-aged adults | 1.52 (1.05-2.26) | 1.25 (0.85-1.85) | 1.16 (0.79-1.73) |
|  |  | Older adults | 0.69 (0.29-1.63) | 0.73 (0.30-1.73) | 0.68 (0.28-1.63) |
| 2-year | Australia | Full group | 1.75 (1.63-1.88) | 1.20 (1.12-1.29) | 1.14 (1.06-1.22) |
|  |  | Male | 1.53 (1.40-1.67) | 1.13 (1.03-1.24) | 1.09 (1.00-1.19) |
|  |  | Female | 2.24 (1.99-2.53) | 1.40 (1.24-1.58) | 1.29 (1.15-1.46) |
|  |  | Young adults | 1.14 (0.98-1.33) | 0.93 (0.80-1.09) | 0.95 (0.81-1.12) |
|  |  | Middle-aged adults | 1.40 (1.29-1.53) | 1.09 (1.00-1.19) | 1.03 (0.95-1.13) |
|  |  | Older adults | 0.90 (0.69-1.17) | 0.88 (0.68-1.15) | 0.82 (0.63-1.08) |
|  | Denmark | Full group | 2.25 (2.12-2.40) | 1.66 (1.56-1.77) | 1.42 (1.33-1.51) |
|  |  | Male | 2.43 (2.23-2.65) | 1.98 (1.81-2.16) | 1.66 (1.52-1.82) |
|  |  | Female | 2.10 (1.92-2.29) | 1.43 (1.31-1.56) | 1.24 (1.13-1.36) |
|  |  | Young adults | 1.00 (0.88-1.15) | 0.91 (0.80-1.05) | 0.91 (0.79-1.05) |
|  |  | Middle-aged adults | 1.98 (1.84-2.13) | 1.45 (1.35-1.56) | 1.25 (1.16-1.35) |
|  |  | Older adults | 3.10 (2.41-4.04) | 3.11 (2.42-4.07) | 2.86 (2.22-3.74) |
|  | UK | Full group | 2.06 (1.58-2.72) | 1.46 (1.12-1.94) | 1.31 (0.99-1.73) |
|  |  | Male | 2.24 (1.58-3.23) | 1.70 (1.20-2.47) | 1.54 (1.08-2.24) |
|  |  | Female | 1.97 (1.31-3.04) | 1.32 (0.87-2.05) | 1.14 (0.75-1.78) |
|  |  | Young adults | 1.49 (0.89-2.68) | 1.37 (0.81-2.48) | 1.40 (0.83-2.54) |
|  |  | Middle-aged adults | 1.49 (1.04-2.16) | 1.06 (0.73-1.55) | 0.91 (0.62-1.33) |
|  |  | Older adults | 0.64 (0.26-1.52) | 0.68 (0.27-1.65) | 0.62 (0.25-1.52) |
| 5-year | Australia | Full group | 2.28 (2.08-2.51) | 1.22 (1.11-1.35) | 1.14 (1.03-1.26) |
|  |  | Male | 1.98 (1.76-2.23) | 1.14 (1.01-1.29) | 1.08 (0.96-1.23) |
|  |  | Female | 2.98 (2.54-3.53) | 1.47 (1.24-1.75) | 1.33 (1.12-1.58) |
|  |  | Young adults | 1.55 (1.24-1.97) | 0.95 (0.76-1.22) | 0.96 (0.76-1.23) |
|  |  | Middle-aged adults | 1.80 (1.61-2.01) | 1.08 (0.96-1.22) | 1.02 (0.90-1.15) |
|  |  | Older adults | 1.23 (0.91-1.71) | 0.95 (0.68-1.34) | 0.87 (0.62-1.22) |
|  | Denmark | Full group | 2.81 (2.63-3.00) | 1.85 (1.73-1.99) | 1.53 (1.42-1.64) |
|  |  | Male | 3.07 (2.80-3.37) | 2.34 (2.12-2.59) | 1.86 (1.68-2.07) |
|  |  | Female | 2.59 (2.36-2.84) | 1.55 (1.40-1.71) | 1.32 (1.19-1.46) |
|  |  | Young adults | 1.43 (1.23-1.67) | 1.02 (0.87-1.21) | 1.00 (0.84-1.19) |
|  |  | Middle-aged adults | 2.56 (2.37-2.76) | 1.52 (1.40-1.65) | 1.29 (1.19-1.40) |
|  |  | Older adults | 3.03 (2.35-3.97) | 3.19 (2.44-4.25) | 2.84 (2.16-3.78) |
|  | UK | Full group | 2.80 (1.97-4.14) | 1.82 (1.25-2.72) | 1.62 (1.12-2.43) |
|  |  | Male | 4.63 (2.72-8.60) | 3.21 (1.85-6.05) | 2.96 (1.70-5.59) |
|  |  | Female | 1.71 (1.07-2.89) | 1.05 (0.64-1.81) | 0.89 (0.54-1.55) |
|  |  | Young adults | 3.48 (1.46-11.37) | 2.14 (0.89-7.03) | 2.08 (0.86-6.84) |
|  |  | Middle-aged adults | 1.97 (1.28-3.18) | 1.16 (0.73-1.91) | 1.00 (0.62-1.65) |
|  |  | Older adults | 1.18 (0.46-3.60) | 1.29 (0.47-4.15) | 1.20 (0.43-3.87) |

Models were adjusted for age, sex, and calendar year at the time of first dispensation of antihypertensive medication. In age-stratified analyses, age was not included as an adjustment variable.

Abbreviations: ADHD, attention-deficit/hyperactivity disorder; CI, confidence interval; OR, odds ratio; UK, United Kingdom; US, United States.

# Table 21. Sensitivity analysis: association between ADHD medication and poor adherence to antihypertensive medication by using the country-specific gap to define discontinuation

| **Year** | **Country** | **Subgroups** | **Model 1**  **OR (95% CI)** | **Model 2**  **OR (95% CI)** | **Model 3**  **OR (95% CI)** |
| --- | --- | --- | --- | --- | --- |
| 1-year | Australia | Full group | 0.97 (0.90-1.05) | 0.78 (0.73-0.85) | 0.75 (0.69-0.81) |
|  |  | Male | 0.90 (0.81-0.99) | 0.78 (0.71-0.87) | 0.76 (0.69-0.85) |
|  |  | Female | 1.11 (0.99-1.24) | 0.81 (0.72-0.91) | 0.76 (0.67-0.85) |
|  |  | Young adults | 0.60 (0.53-0.69) | 0.60 (0.52-0.69) | 0.60 (0.52-0.69) |
|  |  | Middle-aged adults | 0.81 (0.73-0.89) | 0.73 (0.66-0.80) | 0.69 (0.63-0.76) |
|  |  | Older adults | 0.70 (0.49-1.00) | 0.78 (0.54-1.11) | 0.75 (0.52-1.08) |
|  | Denmark | Full group | 1.21 (1.15-1.28) | 0.94 (0.89-0.99) | 0.91 (0.86-0.95) |
|  |  | Male | 1.27 (1.18-1.37) | 1.10 (1.02-1.19) | 1.05 (0.97-1.13) |
|  |  | Female | 1.18 (1.10-1.27) | 0.83 (0.78-0.90) | 0.82 (0.76-0.88) |
|  |  | Young adults | 0.41 (0.37-0.45) | 0.41 (0.37-0.46) | 0.43 (0.38-0.48) |
|  |  | Middle-aged adults | 1.03 (0.96-1.10) | 0.84 (0.78-0.90) | 0.81 (0.75-0.86) |
|  |  | Older adults | 2.85 (2.44-3.34) | 2.93 (2.50-3.43) | 2.96 (2.53-3.46) |
|  | UK | Full group | 1.06 (0.78-1.41) | 0.90 (0.67-1.21) | 0.87 (0.64-1.17) |
|  |  | Male | 1.02 (0.68-1.50) | 0.90 (0.60-1.32) | 0.86 (0.57-1.27) |
|  |  | Female | 1.29 (0.81-2.03) | 0.97 (0.61-1.54) | 0.93 (0.58-1.47) |
|  |  | Young adults | 0.55 (0.32-0.95) | 0.63 (0.36-1.11) | 0.73 (0.41-1.31) |
|  |  | Middle-aged adults | 0.94 (0.64-1.37) | 0.83 (0.56-1.21) | 0.79 (0.53-1.15) |
|  |  | Older adults | 0.71 (0.23-1.87) | 0.83 (0.27-2.17) | 0.81 (0.26-2.14) |
| 2-year | Australia | Full group | 0.96 (0.90-1.03) | 0.75 (0.70-0.81) | 0.72 (0.67-0.77) |
|  |  | Male | 0.84 (0.77-0.92) | 0.72 (0.65-0.79) | 0.70 (0.64-0.77) |
|  |  | Female | 1.18 (1.06-1.30) | 0.83 (0.75-0.92) | 0.77 (0.70-0.86) |
|  |  | Young adults | 0.56 (0.50-0.64) | 0.54 (0.47-0.61) | 0.54 (0.48-0.62) |
|  |  | Middle-aged adults | 0.79 (0.72-0.86) | 0.70 (0.64-0.77) | 0.67 (0.61-0.73) |
|  |  | Older adults | 0.69 (0.49-0.96) | 0.77 (0.54-1.07) | 0.74 (0.52-1.03) |
|  | Denmark | Full group | 1.12 (1.07-1.18) | 0.81 (0.77-0.85) | 0.79 (0.75-0.83) |
|  |  | Male | 1.14 (1.06-1.22) | 0.90 (0.84-0.97) | 0.86 (0.81-0.93) |
|  |  | Female | 1.12 (1.05-1.20) | 0.74 (0.70-0.80) | 0.74 (0.69-0.79) |
|  |  | Young adults | 0.38 (0.35-0.43) | 0.38 (0.34-0.42) | 0.39 (0.35-0.44) |
|  |  | Middle-aged adults | 0.90 (0.84-0.95) | 0.70 (0.66-0.74) | 0.67 (0.63-0.72) |
|  |  | Older adults | 2.57 (2.23-2.98) | 2.72 (2.36-3.15) | 2.77 (2.39-3.20) |
|  | UK | Full group | 0.94 (0.71-1.24) | 0.78 (0.59-1.03) | 0.76 (0.57-1.00) |
|  |  | Male | 0.79 (0.53-1.15) | 0.69 (0.46-1.00) | 0.66 (0.45-0.97) |
|  |  | Female | 1.31 (0.86-2.00) | 0.98 (0.64-1.50) | 0.94 (0.62-1.45) |
|  |  | Young adults | 0.48 (0.29-0.79) | 0.53 (0.31-0.89) | 0.60 (0.35-1.02) |
|  |  | Middle-aged adults | 0.79 (0.54-1.13) | 0.68 (0.47-0.98) | 0.65 (0.45-0.94) |
|  |  | Older adults | 0.79 (0.30-1.91) | 0.84 (0.32-2.04) | 0.83 (0.31-2.01) |
| 5-year | Australia | Full group | 1.02 (0.96-1.09) | 0.76 (0.71-0.80) | 0.72 (0.68-0.76) |
|  |  | Male | 0.89 (0.82-0.96) | 0.72 (0.66-0.78) | 0.70 (0.64-0.76) |
|  |  | Female | 1.25 (1.14-1.36) | 0.82 (0.75-0.90) | 0.77 (0.70-0.84) |
|  |  | Young adults | 0.59 (0.53-0.66) | 0.52 (0.46-0.59) | 0.52 (0.46-0.59) |
|  |  | Middle-aged adults | 0.82 (0.77-0.89) | 0.69 (0.64-0.75) | 0.66 (0.61-0.71) |
|  |  | Older adults | 0.61 (0.44-0.83) | 0.67 (0.48-0.92) | 0.64 (0.46-0.89) |
|  | Denmark | Full group | 0.99 (0.95-1.04) | 0.69 (0.66-0.73) | 0.69 (0.65-0.72) |
|  |  | Male | 0.95 (0.89-1.01) | 0.73 (0.68-0.78) | 0.71 (0.67-0.76) |
|  |  | Female | 1.04 (0.98-1.11) | 0.67 (0.63-0.72) | 0.67 (0.63-0.72) |
|  |  | Young adults | 0.37 (0.33-0.40) | 0.36 (0.32-0.39) | 0.37 (0.33-0.41) |
|  |  | Middle-aged adults | 0.75 (0.71-0.79) | 0.58 (0.54-0.61) | 0.56 (0.53-0.59) |
|  |  | Older adults | 2.15 (1.89-2.46) | 2.39 (2.09-2.73) | 2.43 (2.13-2.79) |
|  | UK | Full group | 0.64 (0.49-0.83) | 0.56 (0.43-0.72) | 0.55 (0.43-0.72) |
|  |  | Male | 0.56 (0.39-0.79) | 0.51 (0.35-0.72) | 0.50 (0.35-0.71) |
|  |  | Female | 0.84 (0.57-1.24) | 0.66 (0.45-0.98) | 0.65 (0.44-0.97) |
|  |  | Young adults | 0.43 (0.27-0.70) | 0.48 (0.29-0.79) | 0.56 (0.34-0.93) |
|  |  | Middle-aged adults | 0.50 (0.35-0.69) | 0.44 (0.31-0.62) | 0.43 (0.30-0.60) |
|  |  | Older adults | 0.49 (0.19-1.15) | 0.53 (0.20-1.25) | 0.53 (0.20-1.24) |

Models were adjusted for age, sex, and calendar year at the time of first dispensation of antihypertensive medication. In age-stratified analyses, age was not included as an adjustment variable.

Abbreviations: ADHD, attention-deficit/hyperactivity disorder; CI, confidence interval; OR, odds ratio; UK, United Kingdom; US, United States.


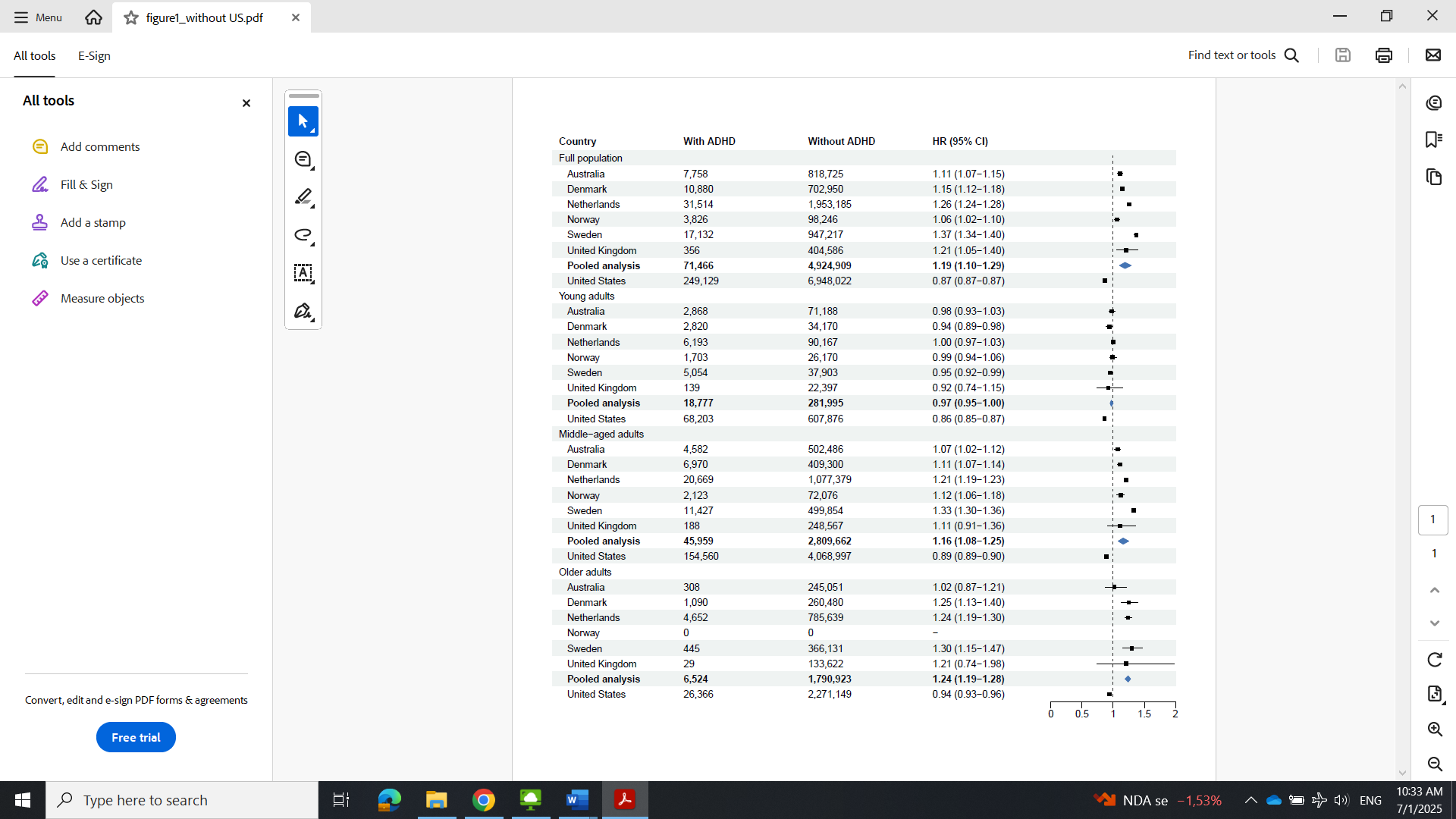


# Fig. S1. Association between ADHD and first discontinuation of antihypertensive medication treatment (pooled estimates excluding US results)

Model 1 was the crude model; Model 2 adjusted for age, sex, and calendar year at the time of first dispensation of antihypertensive medication; Model 3 additionally adjusted for other psychiatric comorbidities. In age-stratified analyses, age was not included as an adjustment variable.

Abbreviations: ADHD, attention-deficit/hyperactivity disorder; CI, confidence interval; HR, hazard ratio; UK, United Kingdom; US, United States.


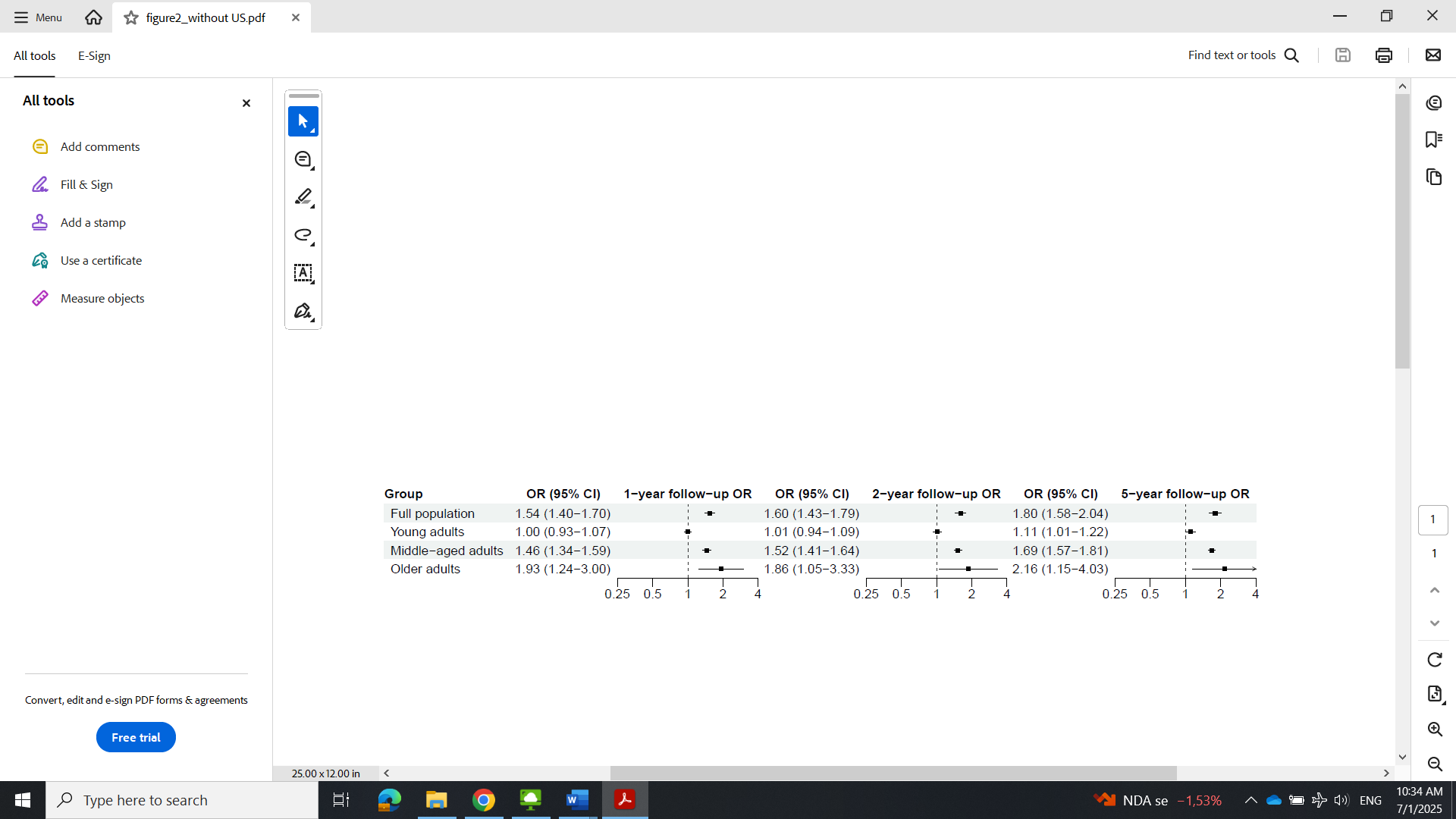


# Fig. S2. Meta-analysis of the association between ADHD and poor adherence to antihypertensive medication (pooled estimates excluding US results)

Model 1 was the crude model; Model 2 adjusted for age, sex, and calendar year at the time of first dispensation of antihypertensive medication; Model 3 additionally adjusted for other psychiatric comorbidities. In age-stratified analyses, age was not included as an adjustment variable.

Abbreviations: ADHD, attention-deficit/hyperactivity disorder; CI, confidence interval; OR, odds ratio; UK, United Kingdom; US, United States.


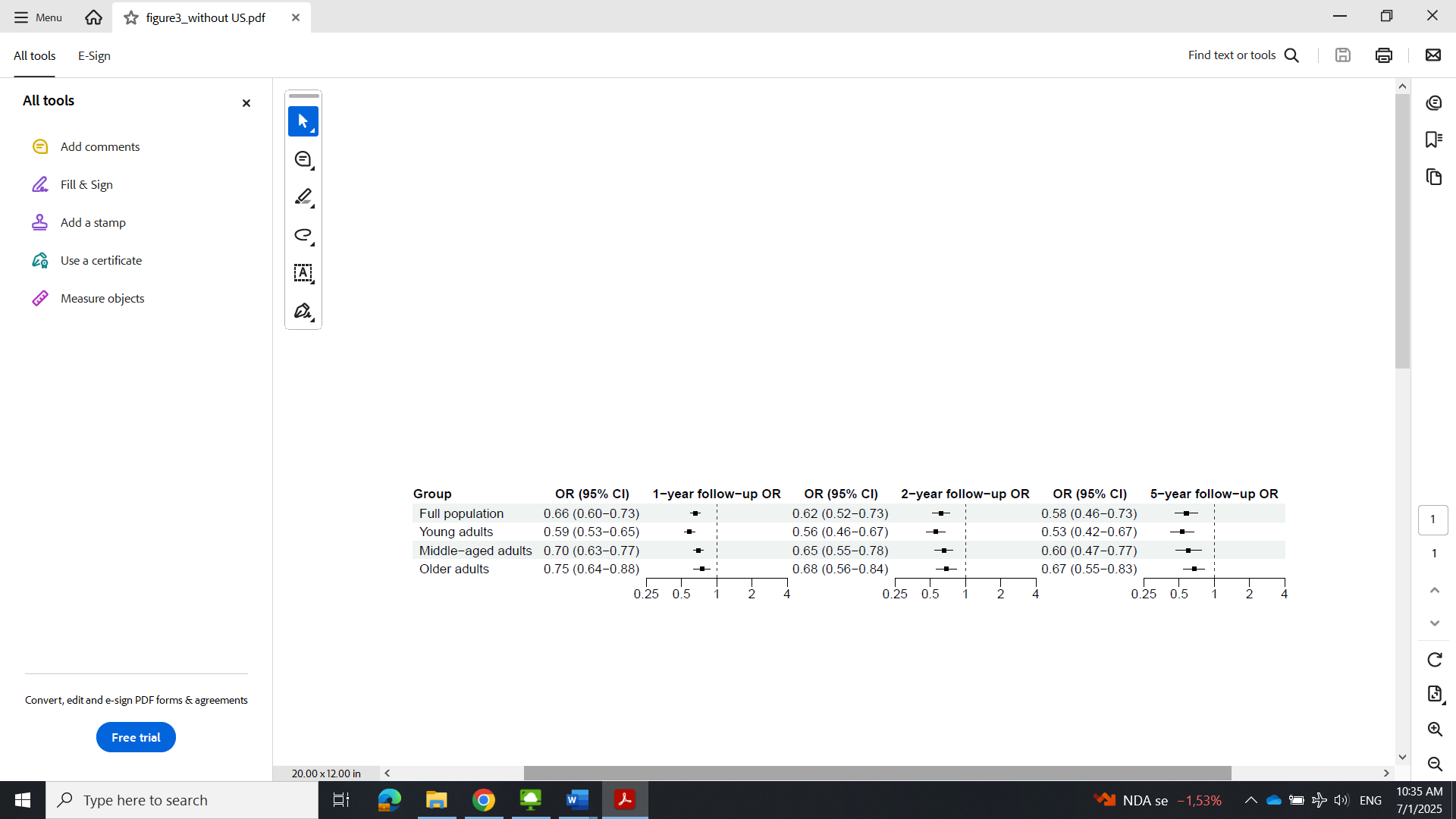


# Fig. S3. Meta-analysis of the association between ADHD medication and poor adherence to antihypertensive medication (pooled estimates excluding US results)

Model 1 was the crude model; Model 2 adjusted for age, sex, and calendar year at the time of first dispensation of antihypertensive medication; Model 3 additionally adjusted for other psychiatric comorbidities. In age-stratified analyses, age was not included as an adjustment variable.

Abbreviations: ADHD, attention-deficit/hyperactivity disorder; CI, confidence interval; OR, odds ratio; UK, United Kingdom; US, United States.
